# Phosphorus Vacancy‐Induced Built‐In Electric Field for Electromagnetic Properties Modulation

**DOI:** 10.1002/advs.202502857

**Published:** 2025-05-23

**Authors:** Yu Zhang, Pengfei Hu, Pei‐Yan Zhao, Bo Cai, Hualong Peng, Shu‐Hao Yang, Martin C. Koo, Chenming Liang, Guang‐Sheng Wang

**Affiliations:** ^1^ Center for Bioinspired Science and Technology Hangzhou International Innovation Institute Beihang University Hangzhou 311115 P. R. China; ^2^ School of Chemistry Beihang University Beijing 100191 P. R. China; ^3^ Research Institute of Aero‐Engine Beihang University Beijing 100191 P. R. China

**Keywords:** built‐in electric fields, defect‐induced polarization, electromagnetic properties, NiCo_0.5_Fe_0.5_P_1‐x_, phosphorus vacancies

## Abstract

Anion vacancy engineering represents an effective strategy to construct built‐in electric fields (BIEFs) for the purpose of modulating electromagnetic (EM) properties. However, the in‐depth and systematic comparative analysis of the effects of various anionic vacancies on defect‐induced polarization is still lacking. In this work, the effects of defect‐induced polarization resulting from group VA anion vacancies, particularly phosphorus vacancies (V_P_), are compared to the anion vacancies of other elements. The EM property modulation mechanisms and quantitative structure‐property relations of NiCo_0.5_Fe_0.5_P_1‐x_ with varying contents of V_P_ are investigated. It is concluded that the high content of V_P_ establishes more intense BIEFs, forming permanent induced dipoles that function as polarization centers, thus enhancing defect‐induced polarization and improving permittivity and dielectric loss. NiCo_0.5_Fe_0.5_P_1‐x3_ with a high content of V_P_ exhibits significant reflection loss (RL) with multi‐band compatibility and wide effective absorption bandwidth (EAB) covering the whole X‐band. This work offers a constructive perspective on the exploration of anionic vacancies from group VA, particularly V_P_, in modulating EM properties. Additionally, it addresses the issue of incompatibility associated with multi‐band strong microwave absorption (MA) and offers a viable strategy for designing advanced metal phosphide MA materials.

## Graphical abstract

The advantages of defect‐induced polarization resulting from group VA anion vacancies, particularly phosphorus vacancies (V_P_) are analyzed in comparison to the anion vacancies of other elements. P atoms possess both plentiful high‐energy unpaired valence electrons in 3p orbitals and moderate nonmetallicity, thus the selection of V_P_ for regulating EM properties presents distinct advantages.

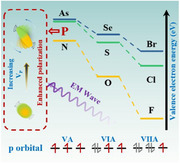

## 1 Introduction

The electromagnetic (EM) properties of materials, particularly permittivity, significantly influence their performance across various fields, including capacitor energy storage,^[^ ^1^ ^]^ electronic devices,^[^ ^2^ ^]^ optical properties,^[^ ^3^ ^]^ EM wave shielding,^[^ ^4^ ^]^ and EM wave absorption.^[^ ^5^, ^6^, ^7^, ^8^ ^]^ Hence, modulating the EM properties of materials from a microscopic perspective represents an effective strategy for optimizing the aforementioned performance of macroscopic materials.^[^ ^9^ ^]^ Constructing built‐in electric fields (BIEFs) at the atomic level inside materials is an effective strategy to enhance polarizability, which is crucial to modulating permittivity. Current research has focused on the construction of BIEFs through multiphase heterogeneous interfaces, which can alter the transport, density and energy band structure of charges, and thus the oscillatory polarization of space charges occurs in interaction with EM waves.^[^ ^10^, ^11^, ^12^ ^]^ For materials with pure phases and perfect crystal structures, their high symmetry restricts the generation of net dipole moments. The introduction of vacancy point defects, especially anion vacancies, can locally disrupt the crystal symmetry, leading to the separation of positive and negative charge centers in the surrounding atoms, where BIEFs can be established to form permanent induced dipoles that act as polarization centers, resulting in defect‐induced polarization under the influence of external EM waves.^[^ ^13^, ^14^, ^15^ ^]^ Moreover, vacancy point defects have the advantage of being quantitatively controllable, thus enabling the investigation of structure‐property relations within the material. However, the in‐depth and systematic comparative analysis of the effects of various anionic vacancies on defect‐induced polarization is still lacking.

The strength of the defect‐induced polarization resulting from anionic vacancies is related to the number of unpaired electrons in the valence electron layer of these atoms, their respective energies, and electron layer positions. The large numbers of high‐energy unpaired valence electrons predispose these atoms to interactions of higher complexity with a greater number of other atoms in their vicinity. Consequently, the loss of nonmetallic atoms leading to the formation of corresponding vacancies results in a stronger defect‐induced polarization when subjected to EM waves, thereby facilitating a more effective modulation of the intrinsic permittivity of the material. Specifically, in comparison to nonmetallic atoms in groups VIA and VIIA in the periodic table, the nonmetallic atoms in group VA of the same period, such as N, P, and As, possess more unpaired electrons in the p orbitals of the valence electron shell (VA‐3, VIA‐2, and VIIA‐1). Additionally, the energies of valence electrons can be calculated using Slater's rule^[^ ^16^ ^]^ and the following equation:

(1) $$E=-\frac{13.6{\left(Z-\sigma \right)}^{2}}{n^{2}}\mathrm{eV}$$

where E represents the electron energy, Z denotes the nuclear charge number, *σ* signifies the shielding constant, and *n* indicates the principal quantum number. The results indicate that the valence electron energies of nonmetallic atoms in group VA are higher than that of the same period in groups VIA and VIIA (Figure S1 and Tables S1, S2, Supporting Information). For various periodic atoms in group VA, the valence electrons of P are in 3p orbitals, which are located farther from the nucleus, rendering the electron cloud more susceptible to deformation and exhibiting stronger polarization ability, compared to the 2p valence electrons of N. Moreover, P exhibits a higher degree of nonmetallic character compared to As, which possesses quasi‐metallic properties. Currently, the application of the anionic vacancy strategy to modulate permittivity primarily has focused on the group VIA elements, such as O,^[^ ^17^ ^]^ S,^[^ ^18^, ^19^ ^]^ and Se^[^ ^20^ ^]^ vacancies. However, based on theoretical analysis, the anionic vacancies of group VA are more favorable in regulating EM properties, in which P atoms possess both plentiful high‐energy unpaired valence electrons in 3p orbitals and moderate nonmetallicity. Thus, the selection of phosphorus vacancies (V_P_) for regulating EM properties presents distinct a dvantages.

Herein, NiCo_0.5_Fe_0.5_P_1‐x_ with varying contents of V_P_ were prepared by adjusting the amount of the phosphorus source. The combination of density functional theory (DFT) calculations and experimental results demonstrates that as the content of V_P_ increases, the polarizability is enhanced and the permittivity correspondingly improves, optimizing impedance matching and enhancing dielectric loss. Meanwhile, the microwave absorption (MA) performance of NiCo_0.5_Fe_0.5_P_1‐x_ were investigated and their scattering properties as radar targets in a real far‐field environment were also simulated. This work investigated the EM properties of metal phosphides with varying V_P_ content, revealing the intrinsic relationship between V_P_ content and EM properties, as well as discussing the underlying atomic‐level mechanisms. Furthermore, it addressed the issue of incompatibility in multi‐band strong MA and provided a viable strategy for the design of advanced metal phosphide MA materials.

## 2 Results and Discussion

### 2.1 Characterizations and DFT Calculations

**Figure 1:**
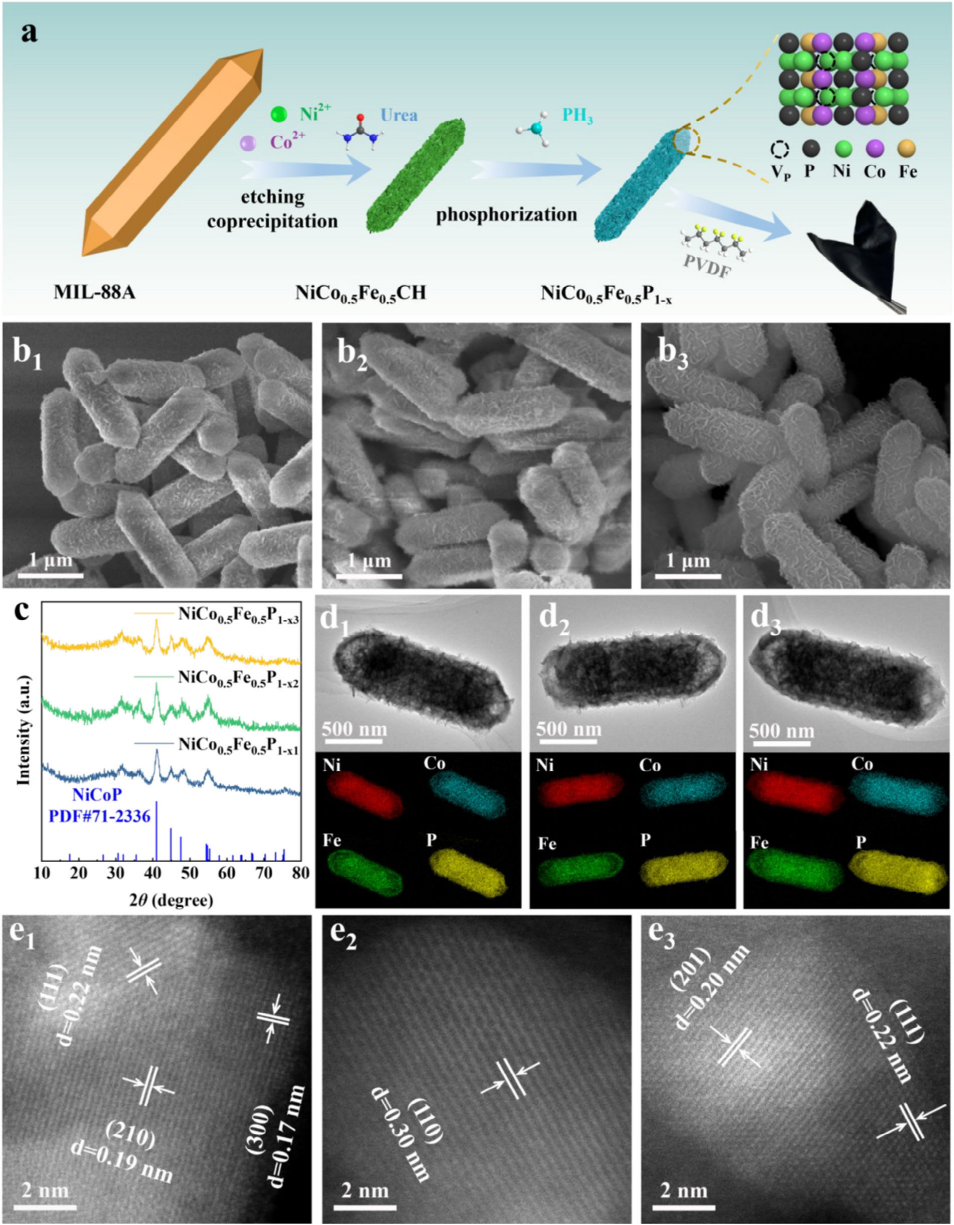
a) Schematic of the preparation process of NiCo_0.5_Fe_0.5_P_1‐x_ and flexible film; SEM images of b_1_) NiCo_0.5_Fe_0.5_P_1‐x1_, b_2_) NiCo_0.5_Fe_0.5_P_1‐x2_, and b_3_) NiCo_0.5_Fe_0.5_P_1‐x3_; c) XRD pattern of NiCo_0.5_Fe_0.5_P_1‐x_; STEM images and corresponding EDS elemental mapping of d_1_) NiCo_0.5_Fe_0.5_P_1‐x1_, d_2_) NiCo_0.5_Fe_0.5_P_1‐x2_, and d_3_) NiCo_0.5_Fe_0.5_P_1‐x3_; HADDF‐STEM images of e_1_) NiCo_0.5_Fe_0.5_P_1‐x1_, e_2_) NiCo_0.5_Fe_0.5_P_1‐x2_, and e_3_) NiCo_0.5_Fe_0.5_P_1‐x3_.

The preparation procedure for NiCo_0.5_Fe_0.5_P_1‐x_ was schematically illustrated in **Figure** **1**a. MIL‐88A was progressively etched to serve as a self‐template during the hydrolysis of Ni^2+^, Co^2+^, and urea, resulting in the breaking of Fe‐O coordination bonds and the release of Fe^3+^. The OH^−^ and CO_3_^2−^ ions, which were slowly released from urea during hydrolysis, coprecipitated with metal ions to form NiCoFe trimetallic carbonate hydroxides (NiCo_0.5_Fe_0.5_CH).^[^ ^21^, ^22^ ^]^ The inorganic phosphorus source, NaH_2_PO_2_, decomposed at 300 °C to produce PH_3_, which phosphorized NiCo_0.5_Fe_0.5_CH in situ to form NiCo_0.5_Fe_0.5_P_1‐x_. The V_P_ content was regulated by modulating the NaH_2_PO_2_ dosage. The phosphatization process was conducted in a sealed environment, wherein PH_3_ gas induced transient high‐pressure conditions, thereby promoting V_P_ formation.^[^ ^23^, ^24^ ^]^ An increase in NaH_2_PO_2_ dosage amplified the transient pressure, which subsequently enhanced the V_P_ content. Based on the V_P_ content, the metal phosphides were labeled as NiCo_0.5_Fe_0.5_P_1‐x1_, NiCo_0.5_Fe_0.5_P_1‐x2_, and NiCo_0.5_Fe_0.5_P_1‐x3_ (x1 < x2 < x3), indicating a progressive increase in V_P_ content.

The morphology of MIL‐88A was observed as hexagonal rods with smooth surfaces. The axial dimension was measured at approximately 7.6 µm, while the radial dimension was approximated to be 1.4 µm (Figure S2a,b, Supporting Information). Following etching and coprecipitation, NiCo_0.5_Fe_0.5_CH exhibited rods with rough surfaces, which were assembled from thin nanosheets, with axial and radial dimensions reduced to approximately 1.8 µm and 0.7 µm, respectively (Figure S2c, Supporting Information). NiCo_0.5_Fe_0.5_P_1‐x_ maintained nearly the same morphology and dimensions as NiCo_0.5_Fe_0.5_CH (Figure 1b).

The X‐ray diffraction (XRD) results displayed that the diffraction peaks of all three samples correspond well to the NiCoP phase (JCPDS no.71−2336) as shown in Figure 1c. Additionally, no other impurity peaks were observed, indicating that the materials consisted of pure phases and that Fe replaced a portion of the Ni or Co sites. The atomic ratio of Ni, Co, and Fe was approximately 2:1:1 based on inductively coupled plasma (ICP) test results (Table S3, Supporting Information), indicating that a portion of the Co was replaced by Fe. The broad diffraction peaks revealed the characterization of small grains in NiCo_0.5_Fe_0.5_P_1‐x_. The diffraction peaks of the precursor MIL‐88A corresponded well with previous reports, indicating the successful synthesis of MIL‐88A (Figure S3a, Supporting Information).^[^ ^25^ ^]^ The diffraction peaks of the intermediate product matched those of Ni_6_Fe_2_(CO_3_)(OH)_16_·H_2_O (JCPDS no.26−1286) and Ni_2_(CO_3_)(OH)_2_·H_2_O (JCPDS no.29−0868) phases, and the shifted peaks at 11.3°, 16.6°, 59.6°, and 61.0° indicated that Co doping into the lattice resulted in both expansion and shrinkage (Figure S3b, Supporting Information).^[^ ^22^ ^]^ This result confirmed that the intermediates were NiCo_0.5_Fe_0.5_CH.

The scanning transmission electron microscopy (STEM) was employed to further elucidate the crystal structure and elemental composition of NiCo_0.5_Fe_0.5_P_1‐x_. The STEM‐energy dispersive X‐ray spectrometry (EDS) results demonstrated that NiCo_0.5_Fe_0.5_P_1‐x_ retained the basic morphology of precursor MIL‐88A, while proving the coexistence of Ni, Co, Fe, and P elements (Figure 1d_1_–d_3_). The high‐angle annular dark‐field (HAADF)‐STEM images exhibited lattice stripes on various crystal planes within the NiCo_0.5_Fe_0.5_P_1‐x_ phase (Figure 1e_1_–e_3_). Specifically, the stripes with crystal plane spacings of 0.17 nm, 0.19 nm, 0.20 nm, 0.22 nm, and 0.30 nm corresponded to the (300), (210), (201), (111), and (110) crystal planes, respectively.

**Figure 2:**
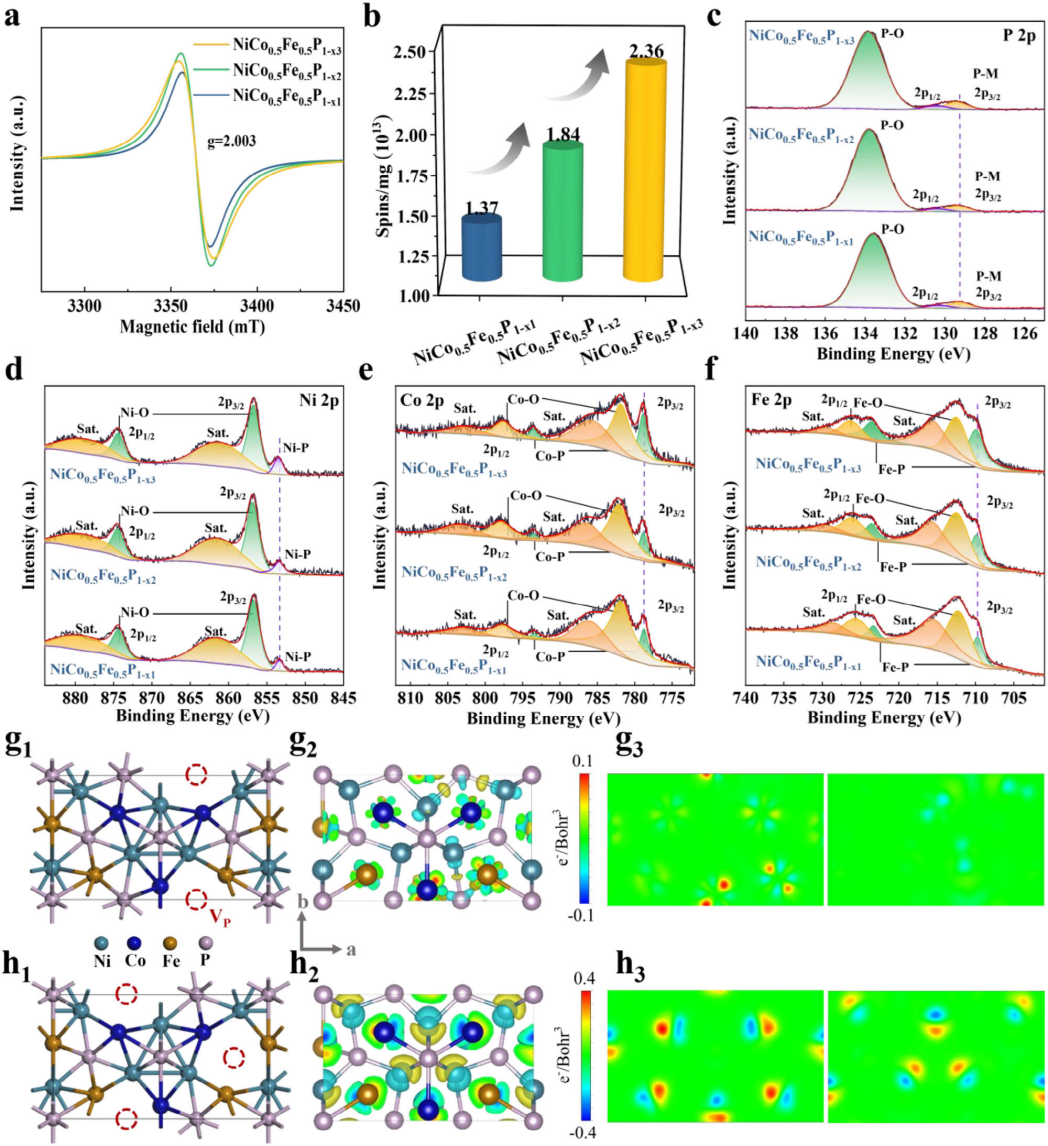
a) EPR spectrum and b) Absolute quantitative results of V_P_ by EPR of NiCo_0.5_Fe_0.5_P_1‐x_; XPS spectra: c) P 2p, d) Ni 2p, e) Co 2p, and f) Fe 2p of NiCo_0.5_Fe_0.5_P_1‐x_; Theoretical structure models of g_1_) NiCo_0.5_Fe_0.5_P_1‐x_‐V_P1_ and h_1_) NiCo_0.5_Fe_0.5_P_1‐x_‐V_P2_; 3D charge density difference of g_2_) NiCo_0.5_Fe_0.5_P_1‐x_‐V_P1_ and h_2_) NiCo_0.5_Fe_0.5_P_1‐x_‐V_P2_; 2D charge density difference of g_3_) NiCo_0.5_Fe_0.5_P_1‐x_‐V_P1_ and h_3_) NiCo_0.5_Fe_0.5_P_1‐x_‐V_P2_ at crystal planes of (001) (left) and (002) (right).

The unpaired spin electrons generated by V_P_ were characterized using electron paramagnetic resonance (EPR). As shown in Figure 2a, a distinct signal was observed at a g value of 2.003, indicating the presence of V_P_.^[^ ^26^ ^]^ Further absolute quantitative results indicated that the number of unpaired spin electrons increases sequentially across the three samples, as shown in **Figure** **2**b. The content levels of V_P_ can be reflected from the number of unpaired spin electrons, with a higher quantity indicating a greater presence of V_P_. Therefore, it can be concluded that the content of V_P_ in the three samples increases progressively. Meanwhile, the U‐I curve results further demonstrated that the presence of V_P_ elevated conductivity due to the rapid charge transport facilitated by the V_P_‐induced BIEFs, therefore suggesting a higher V_P_ content correlated with increased conductivity (Figure S4, Supporting Information).^[^ ^27^, ^28^ ^]^

The X‐ray photoelectron spectroscopy (XPS) technique was employed to analyze the surface chemical composition and to further elucidate the electronic structure of NiCo_0.5_Fe_0.5_P_1‐x_ at varying V_P_ contents. The P 2p spectrum (Figure 2c) exhibited peaks corresponding to P‐M bonds around 129.3 eV, while the peaks for P‐O bonds resulting from residual phosphates during the synthesis process and surface oxidation were observed at 133.84 eV.^[^ ^28^ ^]^ The lower binding energies of the P‐M bond in NiCo_0.5_Fe_0.5_P_1‐x_ compared to P^0^ indicated that P acquired electrons from M, resulting in negatively charged P^δ−^ and positively charged M^δ+^.^[^ ^23^ ^]^ In the Ni 2p (Figure 2d), Co 2p (Figure 2e), and Fe 2p (Figure 2f) spectra, the peaks at low binding energies correspond to M (Ni, Co, or Fe)‐P bonds, while the peaks at high binding energies were attributed to M─O bonds resulting from surface oxidation, with satellite peaks are also observable. The P─O and M─O bonds resulting from surface oxidation process is attributed to the high sensitivity of V_P_ to oxygen, which is caused by the presence of unpaired electrons on their surface.^[^ ^29^ ^]^ As the content of V_P_ increases, the peaks corresponding to P─M and M─P bonds were slightly shifted to higher binding energies, signifying that the increased content of V_P_ resulted in electron accumulation on the M and P atoms.^[^ ^30^ ^]^ The XPS survey spectra, along with the C 1s and O 1s spectra, were presented in Figure S5 (Supporting Information).

The DFT calculations were conducted to elucidate the effect of V_P_ on the electron distribution of the surrounding atoms. The model of NiCo_0.5_Fe_0.5_P, devoid of vacancies, was constructed by doping Fe atoms into the NiCoP crystal structure to replace a part of Co atoms based on the results from ICP analysis. To develop the most accurate model of NiCo_0.5_Fe_0.5_P, the positions for Fe doping were systematically screened. As illustrated in Figure S6 (Supporting Information), model 7 exhibited the lowest energy; therefore, it was selected as the theoretical model for NiCo_0.5_Fe_0.5_P. To simulate the effects of different contents of V_P_ on the electron distribution among the surrounding atoms, two models were developed: one incorporating a single vacancy (NiCo_0.5_Fe_0.5_P_1‐x_‐V_P1_) and the other incorporating two vacancies (NiCo_0.5_Fe_0.5_P_1‐x_‐V_P2_), both based on the NiCo_0.5_Fe_0.5_P model (Figures S7, S8, Supporting Information). The most accurate vacancy models were presented in Figure 2g_1_ and h_1_, and the modeled perspectives from the other two directions were illustrated in Figure S9 (Supporting Information). The charge density difference effectively visualizes the distribution of electrons surrounding the atoms. As depicted in Figure 2g_2_, g_3_, and h_2_, h_3_, V_P_ locally disrupt the crystal structure symmetry and induces the separation of positive and negative charge centers in the surrounding atoms, with an enrichment and depletion on each side. Furthermore, as the number of V_P_ increases, the asymmetric distribution of electrons became more pronounced. This observation was consistent with the previously discussed XPS results. As a result, BIEFs were established to form permanent induced dipoles as polarization centers and exhibited significant polarization relaxation in response to EM waves, accompanied by enhanced EM wave dissipation. Meanwhile, the effect of the BIEFs was more pronounced at higher levels of V_P_ content, further enhancing defect‐induced polarization.

### 2.2 EM Properties of NiCo_0.5_Fe_0.5_P_1‐x_ with Various Contents of V_P_

**Figure 3:**
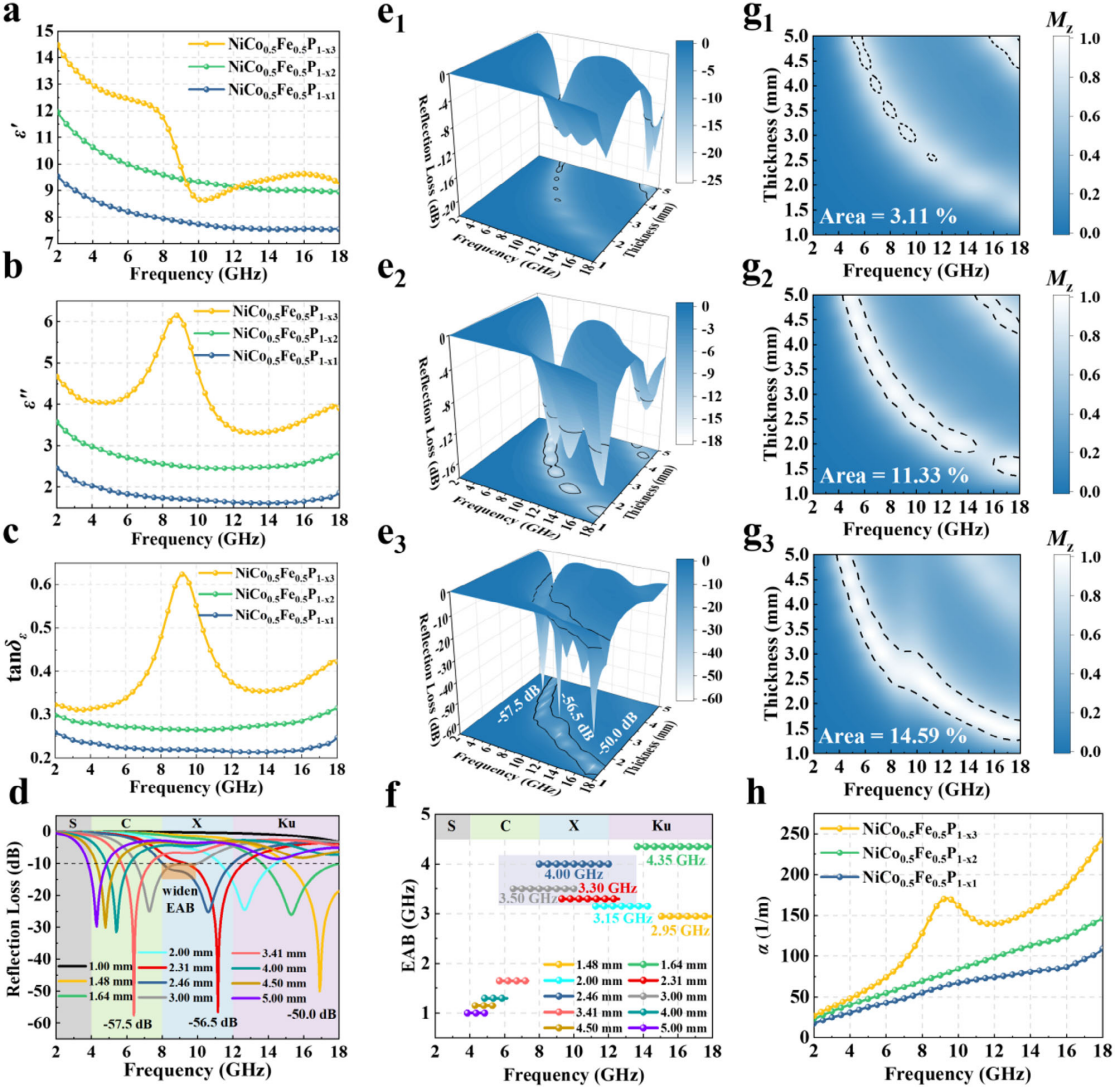
Dielectric characterization: a) real part *ε*′, b) imaginary part *ε*″, and c) dielectric loss tangent tan*δ_ε_* of NiCo_0.5_Fe_0.5_P_1‐x_ with various V_P_ contents; d) 2D RL curves of NiCo_0.5_Fe_0.5_P_1‐x3_; 3D RL curves of e_1_) NiCo_0.5_Fe_0.5_P_1‐x1_, e_2_) NiCo_0.5_Fe_0.5_P_1‐x2_, and e_3_) NiCo_0.5_Fe_0.5_P_1‐x3_; f) EAB values of NiCo_0.5_Fe_0.5_P_1‐x3_; 2D isopach map of the impedance matching coefficient *M_z_* of g_1_) NiCo_0.5_Fe_0.5_P_1‐x1_, g_2_) NiCo_0.5_Fe_0.5_P_1‐x2_, and g_3_) NiCo_0.5_Fe_0.5_P_1‐x3_; h) Attenuation constants *α*.

The EM parameters of polyvinylidene fluoride (PVDF)‐based NiCo_0.5_Fe_0.5_P_1‐x_ with various contents of V_P_ were tested using a vector network analyzer (VNA). The MA properties of a material are dependent on their EM parameters, including relative complex permittivity (*ε_r_ = ε′−*j*ε″*) and relative complex permeability (*µ_r_* = *µ*′−j*µ*″). The real parts (*ε*′ and *µ*′) represent the storage capabilities of electrical and magnetic energy, while the imaginary parts (*ε*″ and *µ*″) denote the ability to dissipate EM energy. As illustrated in **Figure** **3**a,b, both *ε*′ and *ε*″ values exhibit the same incremental trend as the content of V_P_ increases. The parameter *ε*′ is associated with polarization capacity, and the presence of V_P_ induces defect‐induced polarization when interacting with an EM wave. As the content of V_P_ increases, the polarization ability increases in strength, which corresponds to an improvement in *ε*′, as demonstrated in NiCo_0.5_Fe_0.5_P_1‐x1_ (9.5 to 7.5), NiCo_0.5_Fe_0.5_P_1‐x2_ (11.9 to 8.9), and NiCo_0.5_Fe_0.5_P_1‐x3_ (14.5 to 9.3), respectively. This result further corroborates the reliability of the DFT calculations. The parameter *ε*″ is correlated with polarization loss resulting from the polarization process and conduction loss due to leakage currents. Following the rise of V_P_ content, both the polarization ability and conductivity of NiCo_0.5_Fe_0.5_P_1‐x_ are enhanced, resulting in a corresponding improvement in *ε*″, as displayed in NiCo_0.5_Fe_0.5_P_1‐x1_ (2.5 to 1.9), NiCo_0.5_Fe_0.5_P_1‐x2_ (3.6 to 2.8), and NiCo_0.5_Fe_0.5_P_1‐x3_ (4.7 to 3.9), respectively. Notably, the parameter *ε*′ of NiCo_0.5_Fe_0.5_P_1‐x3_ appears to drop steeply, while *ε*″ rises sharply in the frequency range of 8 to 10 GHz, which is characteristic of a typical Debye‐type dielectric relaxation phenomenon.^[^ ^31^ ^]^ The dielectric loss tangent (tan*δ_ε_* = *ε*″/*ε*′) were calculated to assess the ability of the samples to dissipate electrical energy, and the corresponding patterns exhibited the same trend as *ε″* (Figure 3c). The progressive escalation of tan*δ_ε_* is directly linked to the surge of V_P_ content, demonstrating that the increase in V_P_ positively affects microwave loss. To further analyze the dielectric relaxation process and the dielectric loss mechanism of the samples, Cole‐Cole plots were utilized in accordance with the Debye relaxation theory (Figure S10, Supporting Information):^[^ ^32^ ^]^

(2) $${\left(\varepsilon^{\prime }-\frac{\varepsilon_{s}+\varepsilon_{\infty }}{2}\right)}^{2}+{\left(\varepsilon^{\prime \prime }\right)}^{2}={\left(\frac{\varepsilon_{s}-\varepsilon_{\infty }}{2}\right)}^{2}$$

where *ε_s_* and *ε_∞_* are the static permittivity as the frequency approaches 0 and the permittivity in the limit as frequency approaches infinity, respectively. The curves for NiCo_0.5_Fe_0.5_P_1‐x1_ and NiCo_0.5_Fe_0.5_P_1‐x2_ do not exhibited distinct arcs, indicating that the defect‐induced polarization effect was weak at low content of V_P_. In contrast, NiCo_0.5_Fe_0.5_P_1‐x3_ exhibited a prominent standard circular arc in the frequency range of 6 to 12 GHz, representing an intense polarization relaxation process. The high content of V_P_ generates a substantial number of induced dipoles, resulting in significant defect‐induced polarization. The heterogeneous interface between NiCo_0.5_Fe_0.5_P_1‐x_ and PVDF induced interfacial polarization effects.^[^ ^33^ ^]^ Polarization relaxation effects corresponding to different polarization types are observed at various frequencies, and are also reflected by distinct arcs on Cole‐Cole curves.^[^ ^34^ ^]^ The value of *ε′* exhibits an overall gradual decrease with increasing frequency in the 2–18 GHz range, which is consistent with the Debye relaxation theory. Meanwhile *ε*″ also displays a decreasing trend with increasing frequency. Therefore, the corresponding frequency of the interface polarization relaxation peak is expected to be lower than 2 GHz.^[^ ^35^ ^]^ Moreover, the Cole‐Cole curve of NiCo_0.5_Fe_0.5_P_1‐x3_ exhibited a single prominent semicircle corresponding to defect‐induced polarization, indicating the absence of a relaxation process associated with interfacial polarization within the 2–18 GHz range. In addition to polarization loss, conductive loss is also a component of dielectric loss. To further analyze the respective contributions of polarization loss and conductive loss, the U‐I curves of the coaxial rings were experimentally measured, and the conductivity was calculated (Figure S11 and Table S4, Supporting Information).

According to Debye's theory, *ε_c_* ″ can be determined using the following equation:^[^ ^36^ ^]^

(3) $${\varepsilon_{c}}^{\prime \prime }=\frac{\sigma }{\omega \varepsilon_{0}}$$

where *ω* represents angular frequency, *σ* denotes electrical conductivity, while *ε*_0_ is the vacuum dielectric constant (8.85×10^−12^ F m^−1^). The results demonstrated that the conductivity of the three coaxial rings exhibited a sequential increase, accompanied by a gradual rise in *ε_c_* ″. Notably, the absolute value of *ε_c_* ″ is multiple orders of magnitude lower compared to *ε*″, indicating that the conductive loss contributes negligibly to the overall dielectric loss. The values of *µ*′ and *µ*″ for all three samples are approximately 1.0 and 0, respectively, and the magnetic loss tangent (tan*δ_µ_* = *µ*″/*µ*′) values are also approximately 0 (Figure S12, Supporting Information). This indicates that the effect of V_P_ on permeability is minimal, and NiCo_0.5_Fe_0.5_P_1‐x_ exhibits a weak magnetic loss capability. Consequently, the dielectric loss is the primary contributor to MA. The VSM test results indicated that the magnetization intensity of NiCo_0.5_Fe_0.5_P_1‐x_ was exceedingly low (below 1 emu g^−1^), suggesting that the material was nearly non‐magnetic (Figure S13, Supporting Information).

The RL and EAB are key indicators for evaluating the MA performance of materials. The RL values can be calculated based on the following two equations:^[^ ^37^ ^]^

(4) $$\mathrm{RL}=20\log\left|\frac{Z_{in}-Z_{0}}{Z_{in}+Z_{0}}\right|$$

(5) $$Z_{in}=Z_{0}\sqrt{\frac{\mu_{r}}{\varepsilon_{r}}}\tanh\left[j\left(\frac{2f\pi d}{c}\right)\sqrt{\mu_{r}\varepsilon_{r}}\right]$$

where *Z_in_* and *Z_0_* (377 Ω) represent the normalized input impedance and free‐space wave impedance, respectively. *f* is the incident EM wave frequency, *d* denotes the thickness of the absorbers, and *c* signifies the speed of light in vacuum. The EAB is defined as the frequency range in which the RL is less than −10 dB. The RL gradually enhanced with increasing content of V_P_, and NiCo_0.5_Fe_0.5_P_1‐x3_ exhibited optimal RL of −57.5 dB at 6.4 GHz (3.41 mm), −56.5 dB at 11.15 GHz (2.31 mm), and −50.0 dB at 16.9 GHz (1.48 mm), which realized the goal of strong absorption with multi‐band compatibility (Figure 3d,e). The strong absorption capabilities in the C and X bands were precisely due to the enhanced defect‐induced polarization generated within the frequency range of 6 to 12 GHz. The maximum EAB was 4.35 GHz (13.65–18.00 GHz) at a thickness of 1.64 mm, encompassing 72.5% of the Ku‐band. Notably, an EAB of 4.00 GHz (8.00–12.00 GHz) was achieved at a thickness of 2.46 mm, providing full coverage of the X‐band. At a thickness of 3.41 mm, an EAB of 1.65 GHz (5.7–7.35 GHz) also achieved 41.3% coverage within the C‐band (Figure 3f). In general, the EAB tends to narrow as the thickness increases;^[^ ^38^, ^39^, ^40^ ^]^ however, for NiCo_0.5_Fe_0.5_P_1‐x3_, the EAB widened within the 6 to 12 GHz range (purple semi‐transparent rectangular region in Figure 3f), and the widening of the EAB was also evident in the orange semi‐transparent rectangular region in Figure 3d. The enhanced defect‐induced polarization resulting from the high content of V_P_ not only strengthens the RL but also broadens the EAB. The 2D RL graphs of NiCo_0.5_Fe_0.5_P_1‐x1_ and NiCo_0.5_Fe_0.5_P_1‐x2_ were presented in Figure S14 (Supporting Information).

The impedance matching coefficient (*M_z_*) and the attenuation constant (*α*), derived from EM parameter calculations, are two critical factors that determine MA performance. *M_z_* is a prerequisite that dictates how much of the EM wave enters the material. The closer the value of *M_z_* is to 1.0, the larger the portion of EM wave that enters the material without being reflected. It is generally accepted that *M_z_* between 0.8 and 1.0 represents the range in which effective MA is achieved.^[^ ^38^, ^40^, ^41^ ^]^ For samples of finite thickness, the value of *M_z_* is more appropriately calculated by the following equation:^[^ ^41^, ^42^ ^]^

(6) $$M_{z}=\frac{2Z\prime_{in}}{{\left|Z_{in}\right|}^{2}+1}$$

where the *Z*′*_in_* represents the real input impedance. In addition to desirable impedance matching characteristics, the *α* reflecting the EM wave attenuation capability is also an essential factor calculated by the following equation:^[^ ^43^ ^]^

(7) $$\begin{array}{c} \alpha =\frac{\sqrt{2}\pi f}{c}\sqrt{\left(\mu^{\prime \prime }\varepsilon^{\prime \prime }-\mu^{\prime }\varepsilon^{\prime }\right)+\sqrt{{\left(\mu^{\prime \prime }\varepsilon^{\prime \prime }-\mu^{\prime }\varepsilon^{\prime }\right)}^{2}+{\left(\mu^{\prime }\varepsilon^{\prime \prime }+\mu^{\prime \prime }\varepsilon^{\prime }\right)}^{2}}} \end{array}$$

As illustrated in Figure 3e, the effective impedance matching areas of NiCo_0.5_Fe_0.5_P_1‐x_delineated by black dotted lines, reveals an increase as the content of V_P_ rises, with NiCo_0.5_Fe_0.5_P_1‐x3_ exhibiting optimal impedance matching characteristics. It is noteworthy that an increase in V_P_ benefits the optimization of impedance matching when the V_P_ content is maintained within an appropriate range. Excessive V_P_ may further enhance defect‐induced polarization and conductivity, resulting in elevated permittivity. An excessively high permittivity causes impedance mismatch, which is detrimental to MA, underscoring the potential adverse effects of excess V_P_. The *α* values demonstrate a similar upward trend, with NiCo_0.5_Fe_0.5_P_1‐x3_ exhibiting the strongest microwave attenuation capability (Figure 3f). Therefore, the excellent MA performance of NiCo_0.5_Fe_0.5_P_1‐x3_ is deduced to originate from the combination of optimal impedance matching and attenuation capability.

**Figure 4:**
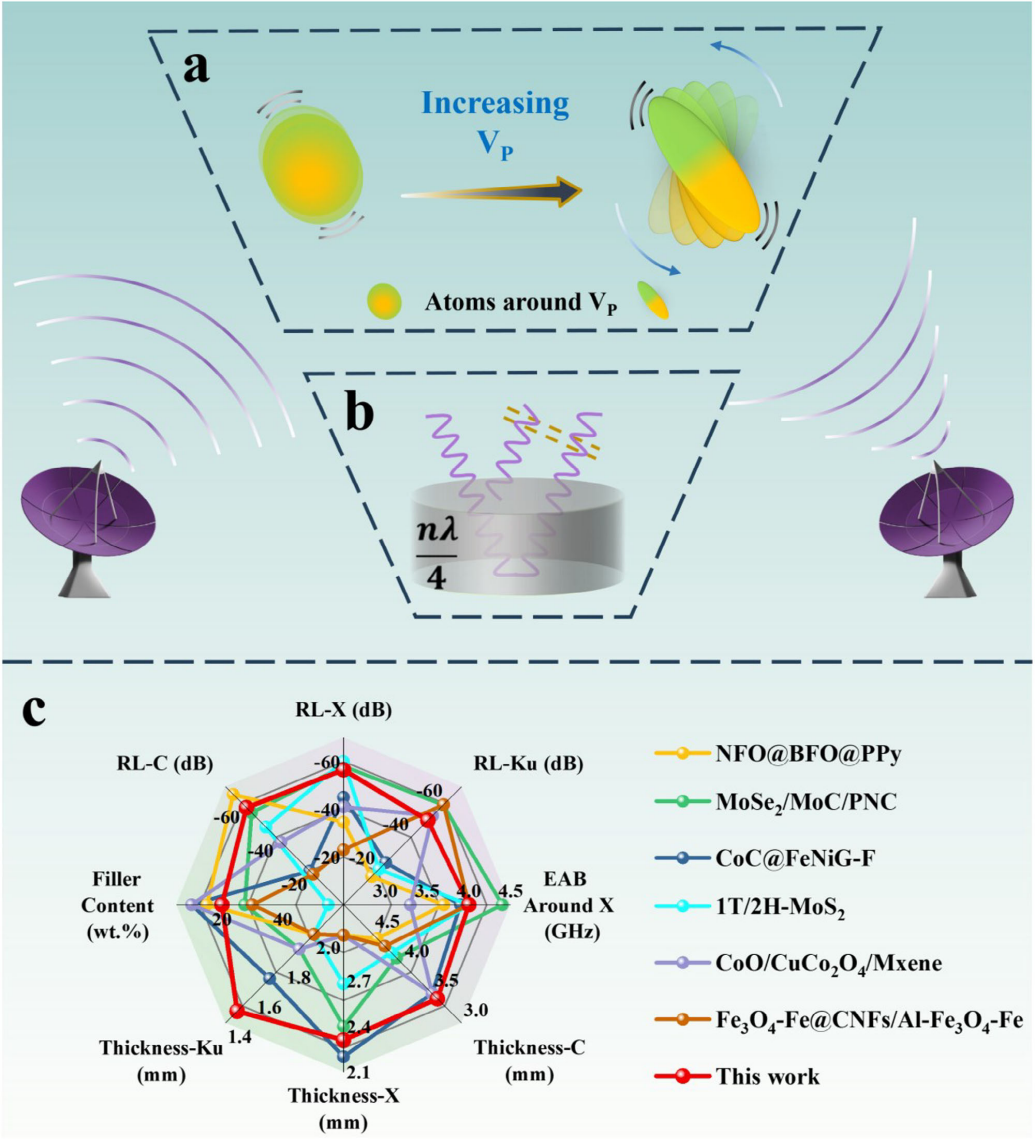
MA mechanism: a) Defect‐induced dipole polarization and b) λ/4 theory; c) Radar graph comparing MA properties.

The underlying MA mechanism of NiCo_0.5_Fe_0.5_P_1‐x_ was illustrated in **Figure** **4**. This mechanism can be divided into two components: (i) microstructure and compositions; (ii) macroscopic geometric structure. From the perspective of microstructure and components, the primary loss mechanism is attributed to the existence of V_P_, which locally disrupts the crystal symmetry, leading to the separation of positive and negative charge centers in the surrounding atoms, allowing for the establishment of BIEFs, resulting in the formation of permanent induced dipoles as polarization centers. As a result, defect‐induced polarization occurs when interacting with EM waves, and as V_P_ content increases the effects of defect‐induced polarization are more pronounced (Figure 4a). Macroscopic geometric structure also plays a significant role in the dissipation of reflected EM waves, and can be investigated based on the λ/4 theory using the following equation:^[^ ^44^ ^]^

(8) $$t_{m}=\frac{n\lambda }{4}=\frac{nc}{4f_{m}\sqrt{\left|\varepsilon_{r}\right|\left|\mu_{r}\right|}}=\left(n=1,3,5\text{…}\right)$$

where *t_m_* denotes the matching thickness, and *f_m_* represents the frequency corresponding to the optimal RL value under the matching thickness. |*ε_r_* | and |*µ_r_* | represent the moduli of the relative complex permittivity and permeability. The λ/4 model creates a phase difference of π between the reflected wave at the front and back of the samples, thereby achieving the extinction of the reflected wave to enhance MA performance (Figure 4b). As shown in Figure S15 (Supporting Information), the experimental values of NiCo_0.5_Fe_0.5_P_1‐x3_ agree well with the theoretical values across the entire frequency range of 2 to 18 GHz, demonstrating the validity of the λ/4 theory. Notably, a deviation exists between the experimental and theoretical values, which is an objective observation. For materials characterized by predominantly dielectric loss, the experimental values exceed the theoretical values, and the greater the value of *tanδ_ε_*, the more pronounced the deviation.^[^ ^41^ ^]^ Moreover, the *M_z_* values at the resonance peaks across various thicknesses are consistently close to 1.0. A comparison of the MA properties between NiCo_0.5_Fe_0.5_P_1‐x3_ and the advanced MA materials reported in recent years was presented in Figure 4c,^[^ ^6^, ^45^, ^46^, ^47^, ^48^, ^49^ ^]^ with the corresponding data listed in Table S5 (Supporting Information). The results indicates that NiCo_0.5_Fe_0.5_P_1‐x3_ exhibits advantages such as strong absorption across multiple bands, a wide EAB in the X‐band, low filler loading, and minimal thickness.

### 2.3 Radar Cross Section (RCS) Simulation Results

**Figure 5:**
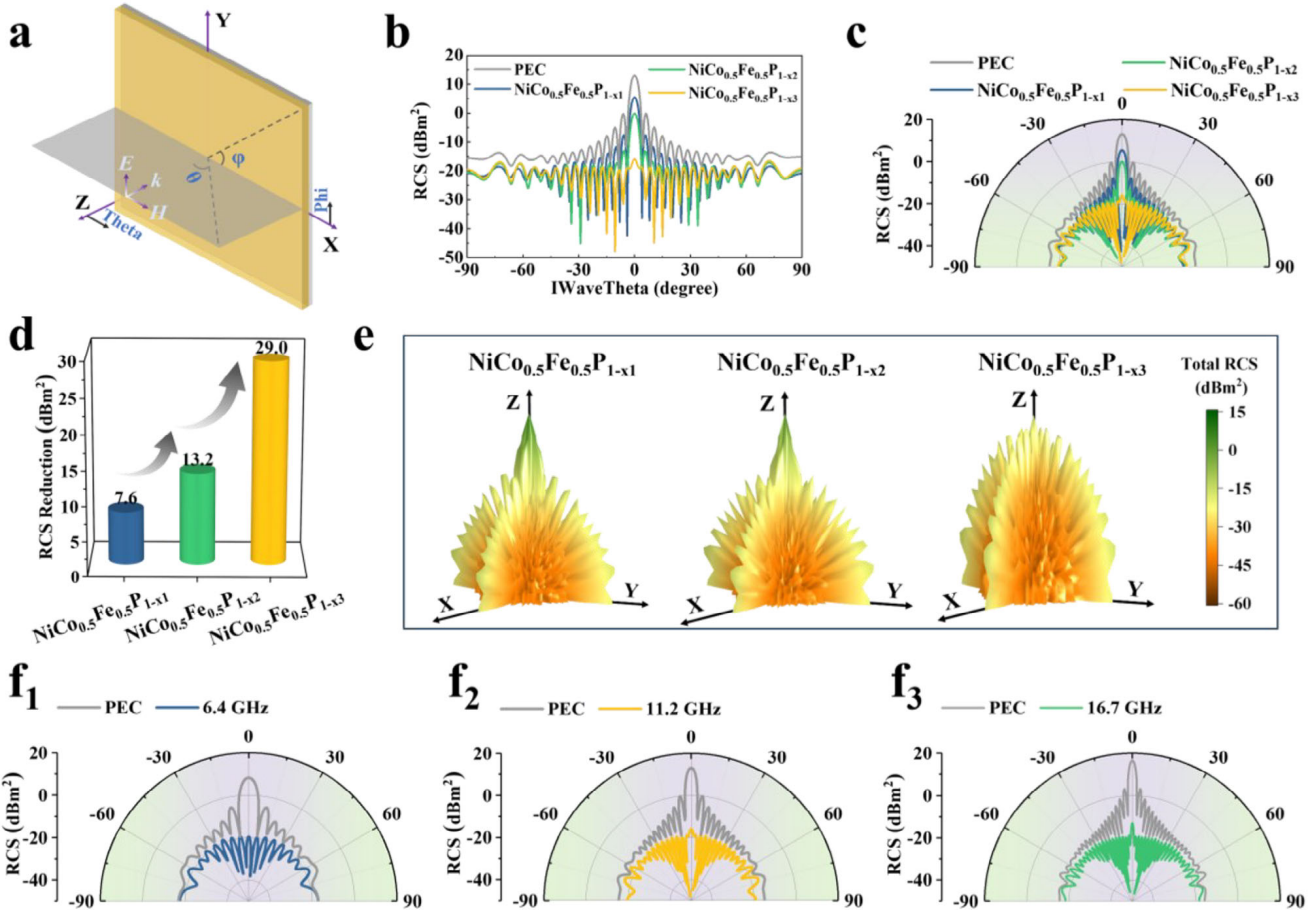
a) RCS simulation model and coordinates; RCS simulation curves in b) rectangular and c) polar coordinates; d) RCS reduction of NiCo_0.5_Fe_0.5_P_1‐x_ with various contents of V_P_; e) 3D radiation patterns of NiCo_0.5_Fe_0.5_P_1‐x_ with various contents of V_P_; RCS simulation curves of NiCo_0.5_Fe_0.5_P_1‐x3_ in polar coordinates at f_1_) 6.4 GHz, f_2_) 11.2 GHz, and f_3_) 16.7 GHz, respectively.

To accurately represent the MA properties of materials in a real far‐field environment, RCS simulations were performed. **Figure** **5**a presented a schematic of the model and its coordinates. Figure 5b,c illustrates the RCS results for NiCo_0.5_Fe_0.5_P_1‐x_ absorber layers with varying V_P_ contents within the angle range of *φ* = 0° and *θ* = −90° to 90°. The data indicates that the RCS value is maximized and the reflection is strongest when the EM wave is incident vertically. As the tilted incidence angle increases, the RCS values gradually decrease and approach a constant value. Moreover, the RCS values of radar‐absorbing materials (RAMs) are all lower than those of pure PEC throughout the wide‐angle range, demonstrating the wide‐angle MA properties. Figure 5d illustrates the RCS reductions of the three RAMs in the most representative main lobe, demonstrating their radar wave attenuation capabilities, which are progressively enhanced compared to pure PEC. This observation was consistent with the trend of the MA properties of the three absorbers discussed previously. The 3D radiation patterns within the angle range of *φ* = 0° to 360° and *θ* = −90° to 90° facilitate a more intuitive observation of radar wave scattering signals from all spatial angles. The comparative results demonstrate significant attenuation of the scattered signal after the application of the RAM, particularly for NiCo_0.5_Fe_0.5_P_1‐x3_ (Figure 5e). Furthermore, the RCS results of the NiCo_0.5_Fe_0.5_P_1‐x3_ absorbing layer at various frequencies and thicknesses were also simulated. The results revealed that it demonstrated excellent radar wave signal attenuation characteristics at 6.4 GHz/3.41 mm, 11.2 GHz/2.31 mm, and 16.7 GHz/1.48 mm (Figure 5f and Figure S16, S17, Supporting Information). This further confirmed that NiCo_0.5_Fe_0.5_P_1‐x3_ possesses strong MA properties across multiple frequency bands.

## 3 Conclusion

This work investigated the advantages of defect‐induced polarization arising from anion vacancies in group VA, particularly V_P_, in comparison to the anionic vacancies of other elements for the modulation of EM properties. The 3p orbitals in the valence electron shell of phosphorus atoms in group VA possess a greater number of unpaired electrons and higher energies, resulting in stronger defect‐induced polarization when subjected to EM waves after the formation of V_P_. Building on this insight, a series of NiCo_0.5_Fe_0.5_P_1‐x_ with varying V_P_ contents were rationally synthesized using self‐templated etching, coprecipitation, and phosphorization methods. The combination of DFT calculations and experimental results concludes that the high content of V_P_ drastically disrupts the local crystal structure symmetry and exacerbates the separation of positive and negative charge centers in the surrounding atoms, which establishes more intense BIEFs to form permanent induced dipoles that behave as polarization centers, thus enhancing defect‐induced polarization, improving permittivity and dielectric loss. NiCo_0.5_Fe_0.5_P_1‐x3_ with high content levels of V_P_ exhibited strong RL with multi‐band compatibility (‐57.5 dB at 6.4 GHz, −56.5 dB at 11.15 GHz, and −50.0 dB at 16.9 GHz) and EAB covering the entire X‐band. NiCo_0.5_Fe_0.5_P_1‐x3_ also demonstrated strong radar wave attenuation performance across multiple frequency bands, with a maximum RCS of 29.6 dBm^2^. This work investigates the relationship between the content of V_P_ and the EM properties of metal phosphides, while also revealing the underlying mechanisms at the atomic level. Additionally, it addresses the issue of incompatibility in multi‐band strong MA and offers a viable strategy for designing advanced metal phosphide MA materials.

## Conflict of Interest

The authors declare no conflict of interest.

## Supporting information



Supporting Information

## Data Availability

Research data are not shared.
