# Supplementary material for: Phosphorus Vacancy‐Induced Built‐In Electric Field for Electromagnetic Properties Modulation
Source: Adv Sci (Weinh). 2025 May 23;12(30):e02857. doi: 10.1002/advs.202502857 (PMC12376502; doi:10.1002/advs.202502857)
Supplement: Supplementary file 1 — Supporting Information [file ADVS-12-e02857-s001.docx]

Supplementary Information

**Phosphorus Vacancy-Induced Built-In Electric Field for Electromagnetic Properties Modulation**

*Yu Zhang, Pengfei Hu, Pei-Yan Zhao, Bo Cai, Hualong Peng, Shu-Hao Yang, Martin C. Koo, Chenming Liang, Guang-Sheng Wang^*^*

Y. Zhang, P. Y. Zhao, B. Cai, H. Peng, S. H. Yang, M. C. Koo, C. Liang, Prof. G. S. Wang

Center for Bioinspired Science and Technology, Hangzhou International Innovation Institute, Beihang University

Hangzhou 311115, P.R. China

E-mail: wanggsh@buaa.edu.cn

Y. Zhang, P. Y. Zhao, B. Cai, H. Peng, S. H. Yang, M. C. Koo, C. Liang, Prof. G. S. Wang

School of Chemistry, Beihang University

Beijing 100191, P.R. China

Prof. P. Hu

Research Institute of Aero-Engine, Beihang University

Beijing 100191, China

Y. Zhang, P. Hu, P. Y. Zhao contributed equally to this work

**Materials and Methods**

**Materials**

All reagents used in the experiment were of analytically pure grade and no further purification was required. Ferric chloride hexahydrate (FeCl_3_·6H_2_O, 99%) and nickel nitrate hexahydrate (Ni(NO_3_)_2_·6H_2_O, 99%) were bought from Xilong Scientific Co., Ltd (Shantou, China). Fumaric acid (C_4_H_4_O_4_, 99.5%), cobalt nitrate hexahydrate (Co(NO_3_)_2_·6H_2_O, 99%), urea (CH_4_N_2_O, 99%), and sodium hypophosphite (NaH_2_PO_2_, 99%) were bought from Shanghai Macklin Biochemical Co., Ltd (Shanghai, China). Anhydrous ethanol (C_2_H_6_O, 99.7%) was purchased from Modern Oriental (Beijing) Technology Development Co., Ltd (Beijing, China). Nitrogen (N_2_, high-purity) was purchased from Beijing Qianxi Jingcheng Gas Co., Ltd (Beijing, China). Polyvinylidene difluoride (PVDF) was obtained from Suzhou Sinero Technology Co., Ltd (Suzhou, China).

**Synthesis of MIL-88A**

MIL-88A was synthesized by an oil bath method, detailed as follows. First, FeCl_3_·6H_2_O (2.703 g, 10 mmol) was dissolved in ultrapure water (50 mL) while being magnetically stirred for 10 min, resulting in solution A. Subsequently, fumaric acid (1.161 g, 10 mmol) was dispersed in ultrapure water (50 mL) while being magnetically stirred for 10 min, yielding solution B. Solutions A and B were mixed and magnetically stirred for 30 min to obtain solution C. Next, solution C was heated in an oil bath at 100 °C for 2 h. Finally, the products were washed by centrifugation (5000 r min^-1^, 3 min) with ultrapure water and anhydrous ethanol three times, and then dried in an oven at 60 °C overnight.

**Preparation of NiCo_0.5_Fe_0.5_CH**

NiCo_0.5_Fe_0.5_CH was prepared by an oil bath method. First, MIL-88A (0.11 g) was dispersed in anhydrous ethanol (60 mL) under ultrasonication for 10 min to obtain solution D. Co(NO_3_)_2_·6H_2_O (0.75 g, 2.6 mmol), Ni(NO_3_)_2_·6H_2_O (0.75 g, 2.6 mmol), and urea (1.00 g, 16.7 mmol) were dissolved in ultrapure water (40 mL) by magnetically stirred for 10 min to obtain solution E. Solutions D and E were mixed and sonicated for 10 min to obtain solution F. Then, the solution F was heated in an oil bath at 95 °C for 10 h. Next, the precipitate of NiCo_0.5_Fe_0.5_CH was obtained by centrifuging (1000 r min^-1^, 3 min) and wash with ultrapure water and anhydrous ethanol three times, respectively, and dried in an oven at 60 °C overnight.

**Fabrication of NiCo_0.5_Fe_0.5_P_1-x_**

NiCo_0.5_Fe_0.5_P_1-x_ was fabricated through a single-step pyrolysis process. Initially, NiCo_0.5_Fe_0.5_CH (0.1 g) and NaH_2_PO_2_ (1.0 g, 11.4 mmol/1.5 g, 17.0 mmol/2.0 g, 22.7 mmol) were separated in a porcelain combustion boat and sealed. Subsequently, the mixture was pyrolyzed in a tube furnace under a nitrogen atmosphere at 300 °C for 2 h with a heating rate of 2 °C min^-1^. Upon cooling to room temperature, the samples of NiCo_0.5_Fe_0.5_P_1-x_ were obtained, and labeled as NiCo_0.5_Fe_0.5_P_1-x1_, NiCo_0.5_Fe_0.5_P_1-x2_, and NiCo_0.5_Fe_0.5_P_1-x3_, respectively (x1<x2<x3).

**Fabrication of NiCo_0.5_Fe_0.5_P_1-x_/PVDF coaxial rings**

The total mass of NiCo_0.5_Fe_0.5_P_1-x_ and PVDF was 0.12 g. The filler content of NiCo_0.5_Fe_0.5_P_1-x_ was 20 wt.% (0.024 g). Firstly, a quantity of NiCo_0.5_Fe_0.5_P_1-x_ and PVDF was dispersed by ultrasound in DMF (5 mL) for 30 min. Subsequently, the homogeneous dispersion was transferred to an oven to evaporate at 90 °C for 4 h to obtain a flexible black film. Finally, the flexible black film was placed into a special mold and pressed at 220 °C and 5 MPa for 15 min, followed by natural cooling to room temperature to obtain the coaxial ring (Φ_out_=7.00 mm and Φ_in_=3.04 mm).

**Material characterization**

The crystal structure and phases of the materials were analyzed using by an X-ray diffraction instrument (XRD, Shimadzu LabX XRD-6000) employing Cu Kα radiation at a scanning rate of 3° min^−1^. The microscopic morphology and structure of the materials were visualized by scanning electron microscopy (SEM, Quanta 250 FEG) and scanning transmission electron microscopy (STEM, Thermofisher Spectra 300). The elemental composition and spatial distribution were characterized via energy dispersive X-ray spectrometry (EDS, Super X EDS detector). The V_P_ were characterized by electron paramagnetic resonance (EPR, Bruker ELEXSYS-II E500). The elemental electronic structure and valence information on material surfaces were characterized by X-ray photoelectron spectroscopy (XPS, Thermo Scientific ESCALAB 250Xi) with Al Kα radiation. The content of metallic elements was tested by Inductively Coupled Plasma Optical Emission Spectrometry (ICP-OES, Agilent 7700). The U-I curves of the pure samples and coaxial rings were measured by a semiconductor analyzer (Keithley 2400), and the dimensions of the rectangular pure sample were 15.8×7.6×2.1 mm^3^. The magnetic properties were tested by vibrating sample magnetometer (VSM, LakeShore 7404). The EM parameters of the coaxial rings were measured with a coaxial approach using a vector network analyzer (VNA, Agilent TE5071C).

**DFT calculation**

The charge density difference and system energy calculations were conducted using density functional theory within the framework of the projector augmented wave method. For the exchange-correlation potential, the generalized gradient approximation proposed by Perdew, Burke, and Ernzerhof was utilized. A cut-off energy of 400 eV was established for the plane wave expansion to ensure adequate accuracy in the calculations. The energy criterion for the iterative solution of the Kohn-Sham equations was set to 10^-6^ eV, facilitating reliable convergence of energy values. Brillouin zone integration was conducted at the Gamma point with a k-mesh grid of 2×4×6. All structures underwent geometric optimization, with the optimization process was deemed successful when the residual forces on the atoms fell below 0.01 eV/Å. This rigorous methodology confirmed that the systems attained their minimum energy configurations, thereby providing a robust foundation for analyzing the electronic properties and stability of the materials under investigation.

**RCS simulation**

The model of RCS simulation consisted of a two-layer square plate in the XOY plane, featuring a radar absorbing material (RAM, 180×180 mm^2^) in the upper layer and a perfect electrical conductor (PEC, 180×180×1 mm^3^) in the lower layer. The excitation was configured as a linearly polarized plane EM wave incident along the Z axis, with the *E* vector oriented along the Phi direction. The radiation boundary was selected as the boundary condition. For the RCS comparison of NiCo_0.5_Fe_0.5_P_1-x_ with varying V_P_ contents, the detection frequency in the far field was set to 11.2 GHz, and the thickness of the RAM layer was established at 2.31 mm. The RCS simulation conditions for NiCo_0.5_Fe_0.5_P_1-x3_ at various detection frequencies and absorbing layer thicknesses were 6.4 GHz/3.41 mm, 11.2 GHz/2.31 mm, and 16.7 GHz/1.48 mm, respectively. All RCS results were derived from monostatic simulations, and the values can be calculated using the following equation:^[S1]^

$\text{σ(dB}\text{m}^{\text{2}}\text{)=10}\log\left( \frac{\text{4π}\text{S}}{\text{λ}^{\text{2}}}\text{∙}\left| \frac{\text{E}_{\text{s}}}{\text{E}_{\text{i}}} \right|^{\text{2}} \right)$ (S1)

Where *λ* denotes the wavelength of EM wave, *S* represents the area of the model, *E*_s_ and *E*_i_ are the electric field intensities of the scattered and incident waves, respectively.

**Supplementary Figures and Tables**

**
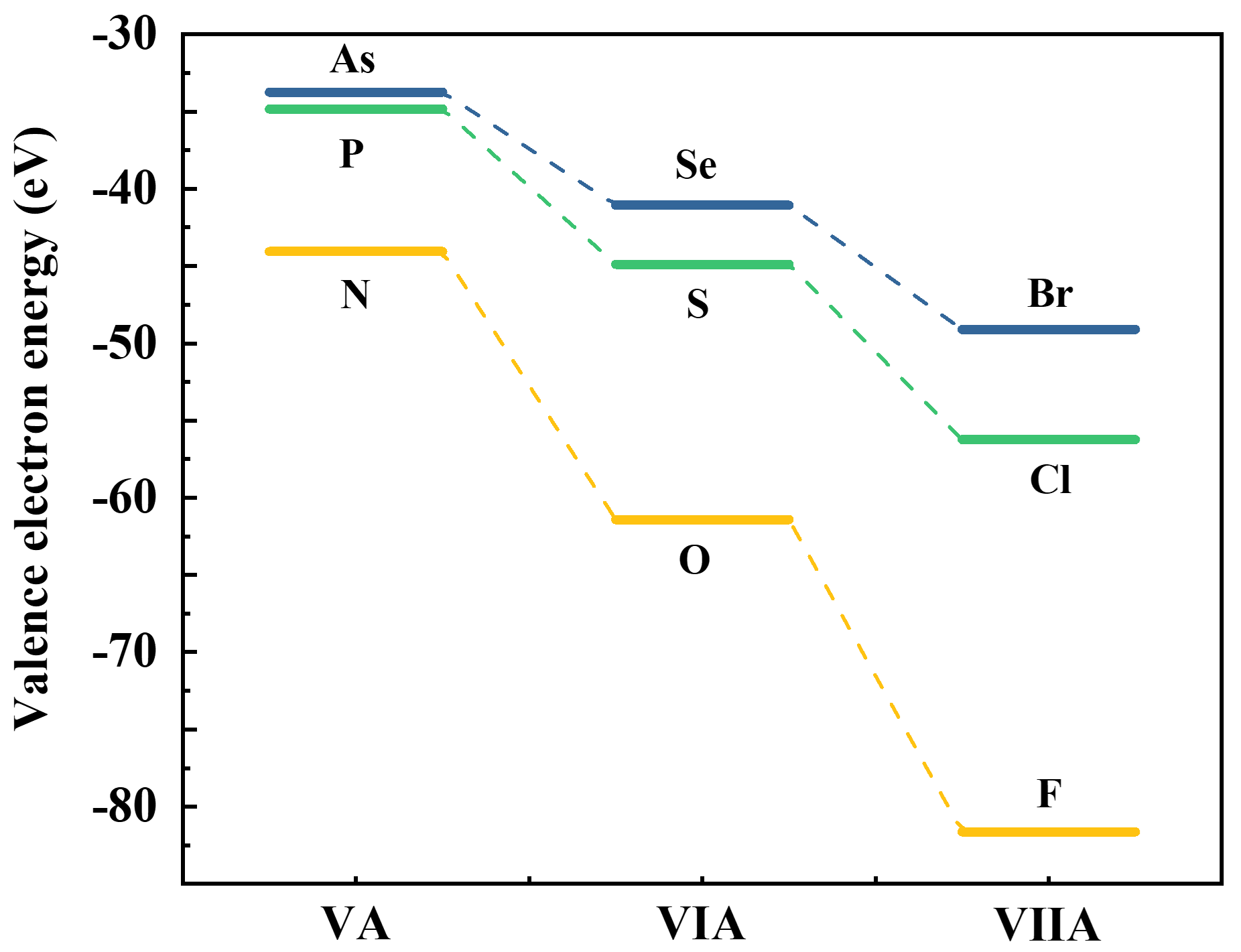
**

**Figure S1.** Valence electron energy (eV) for some nonmetallic atoms of the groups VA, VIA, and ⅦA.


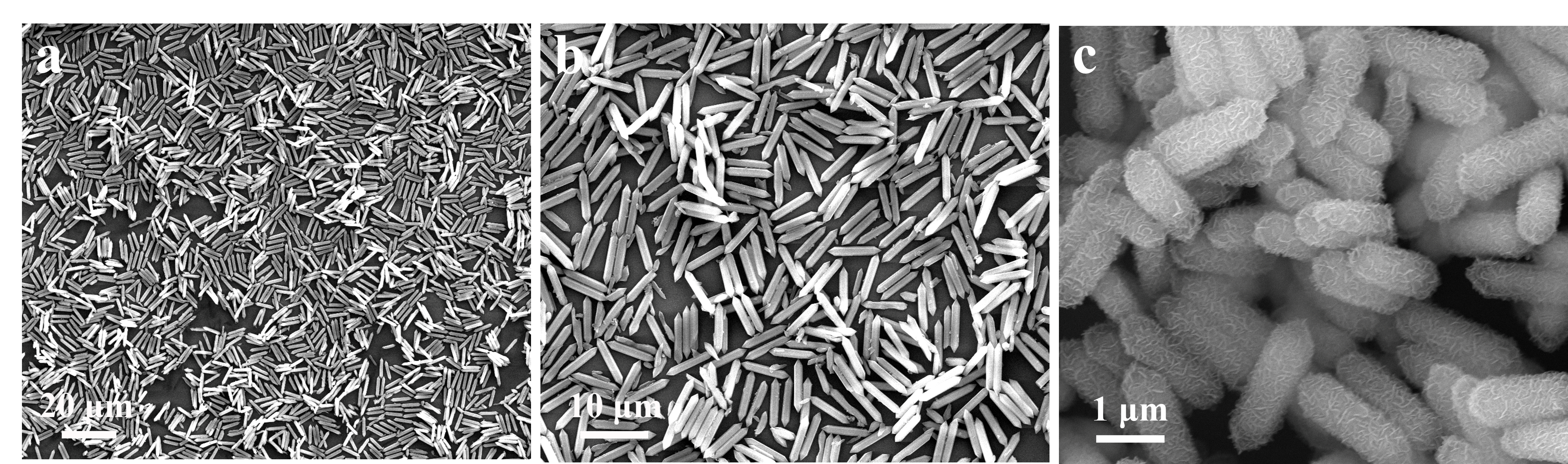


**Figure S2.** SEM images of a, b) MIL-88A and c) NiCo_0.5_Fe_0.5_CH.


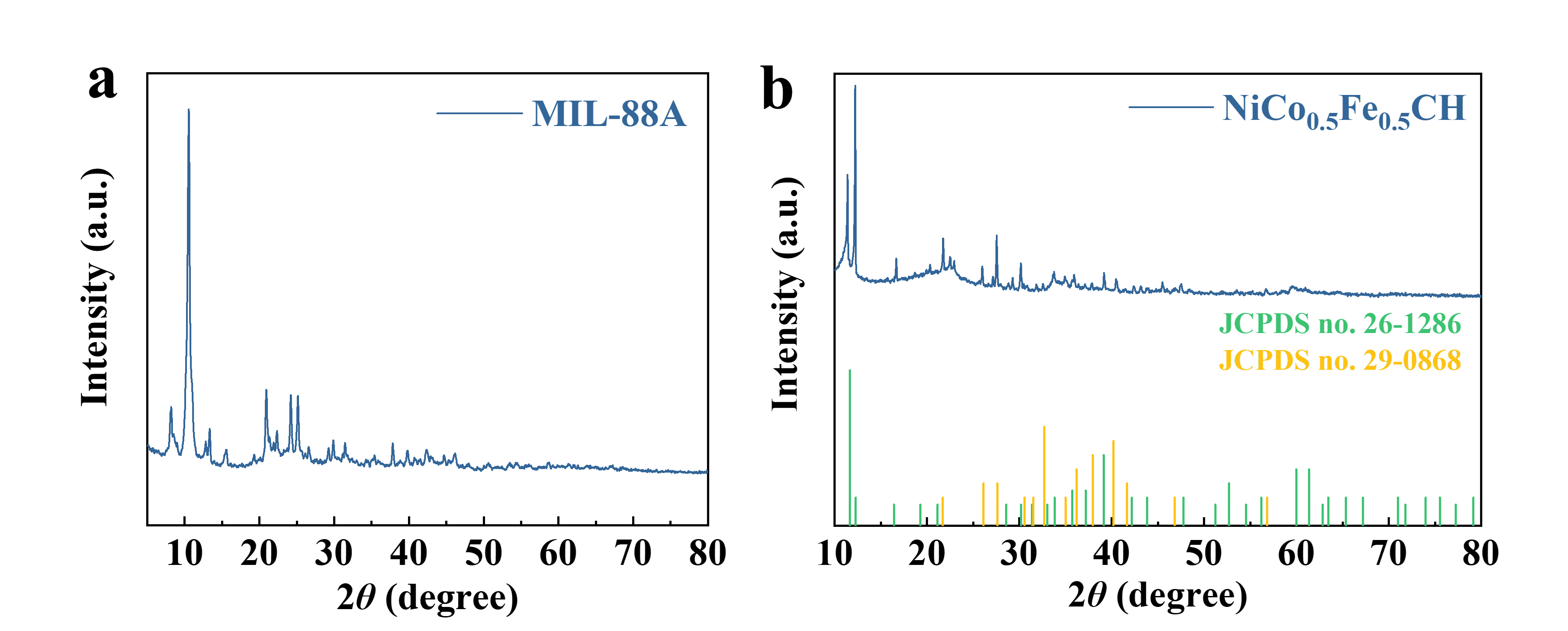


**Figure S3.** XRD patterns of a) MIL-88A and b) NiCo_0.5_Fe_0.5_CH.

**
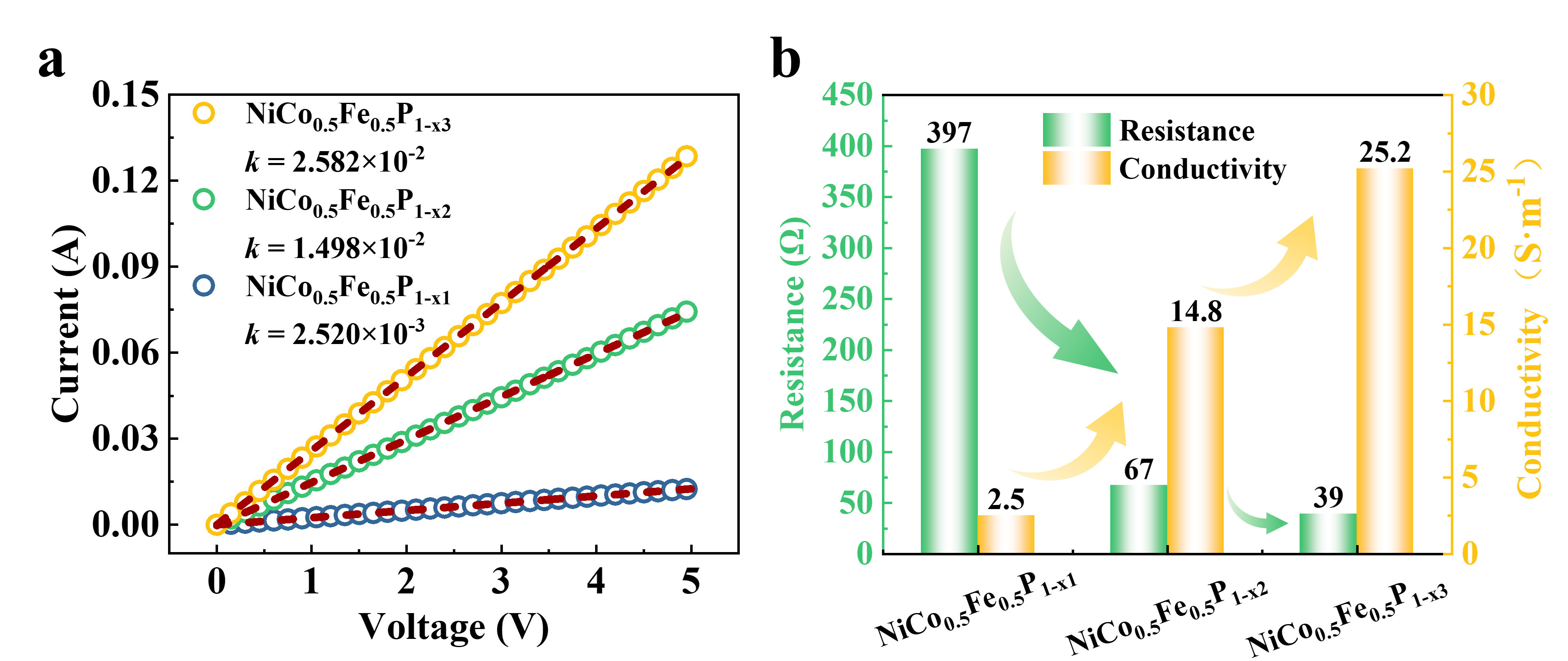
**

**Figure S4.** a) U-I curves and b) Resistance and conductivity of NiCo_0.5_Fe_0.5_P_1-x_.

**
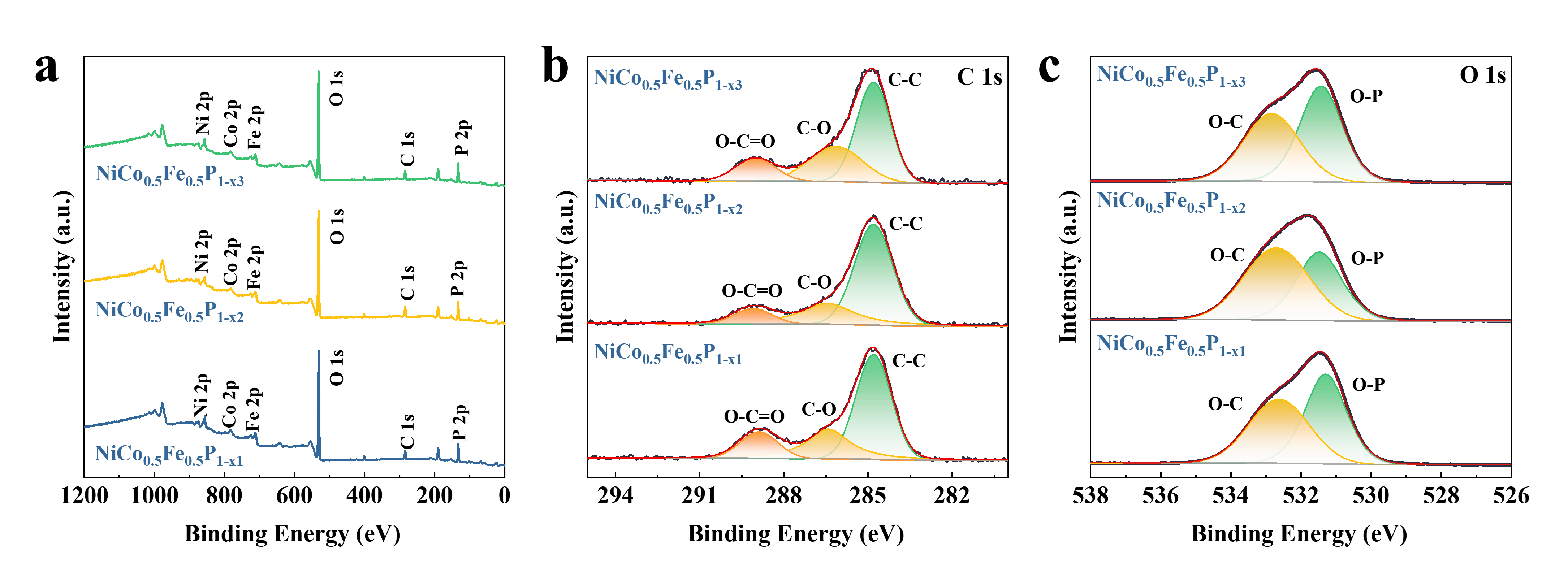
**

**Figure S5.** XPS spectra of NiCo_0.5_Fe_0.5_P_1-x_: a) survey spectrum, b) C 1s, and c) O 1s.


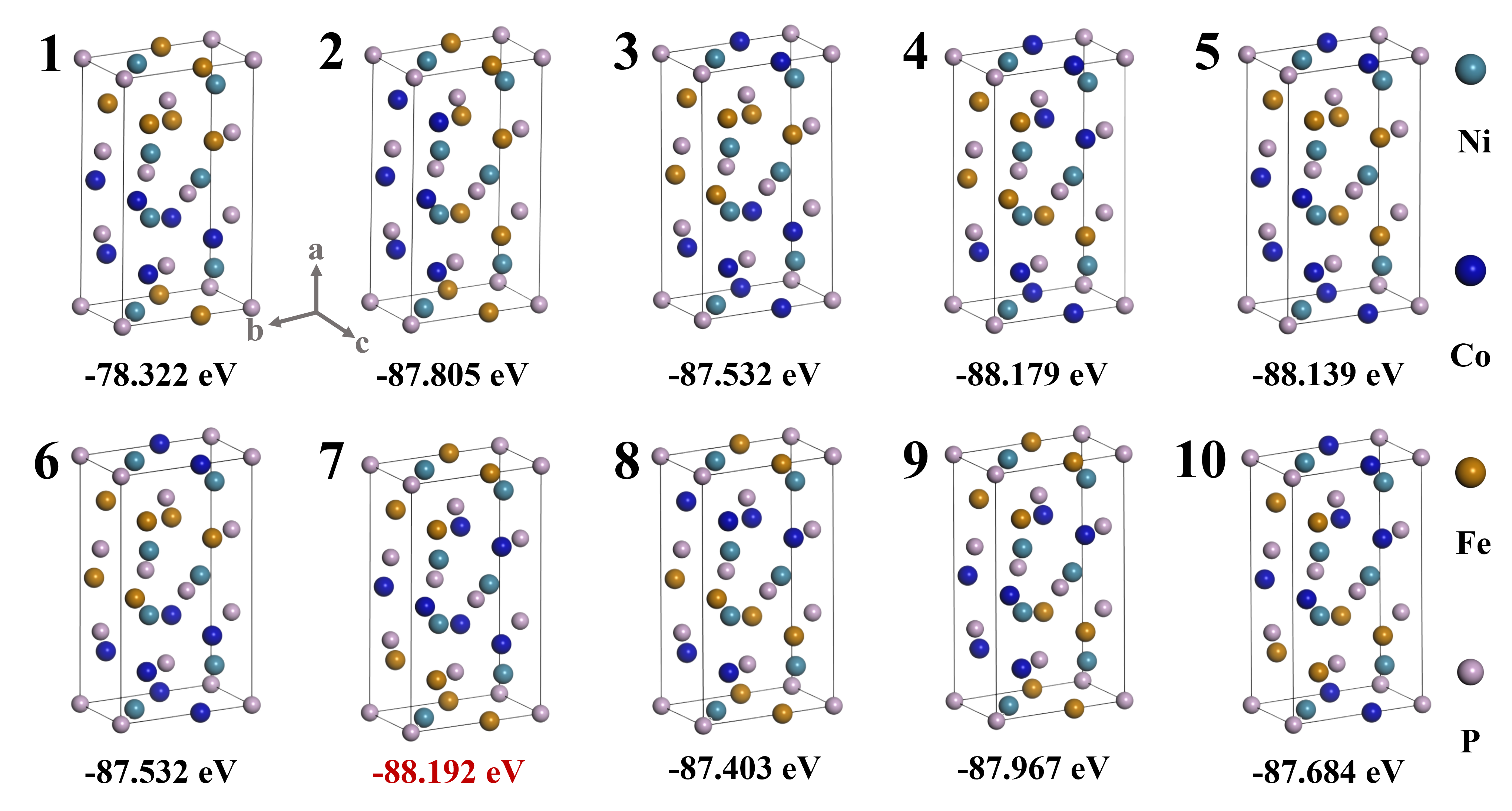


**Figure S6.** Theoretical structure models of NiCo_0.5_Fe_0.5_P without V_P_.


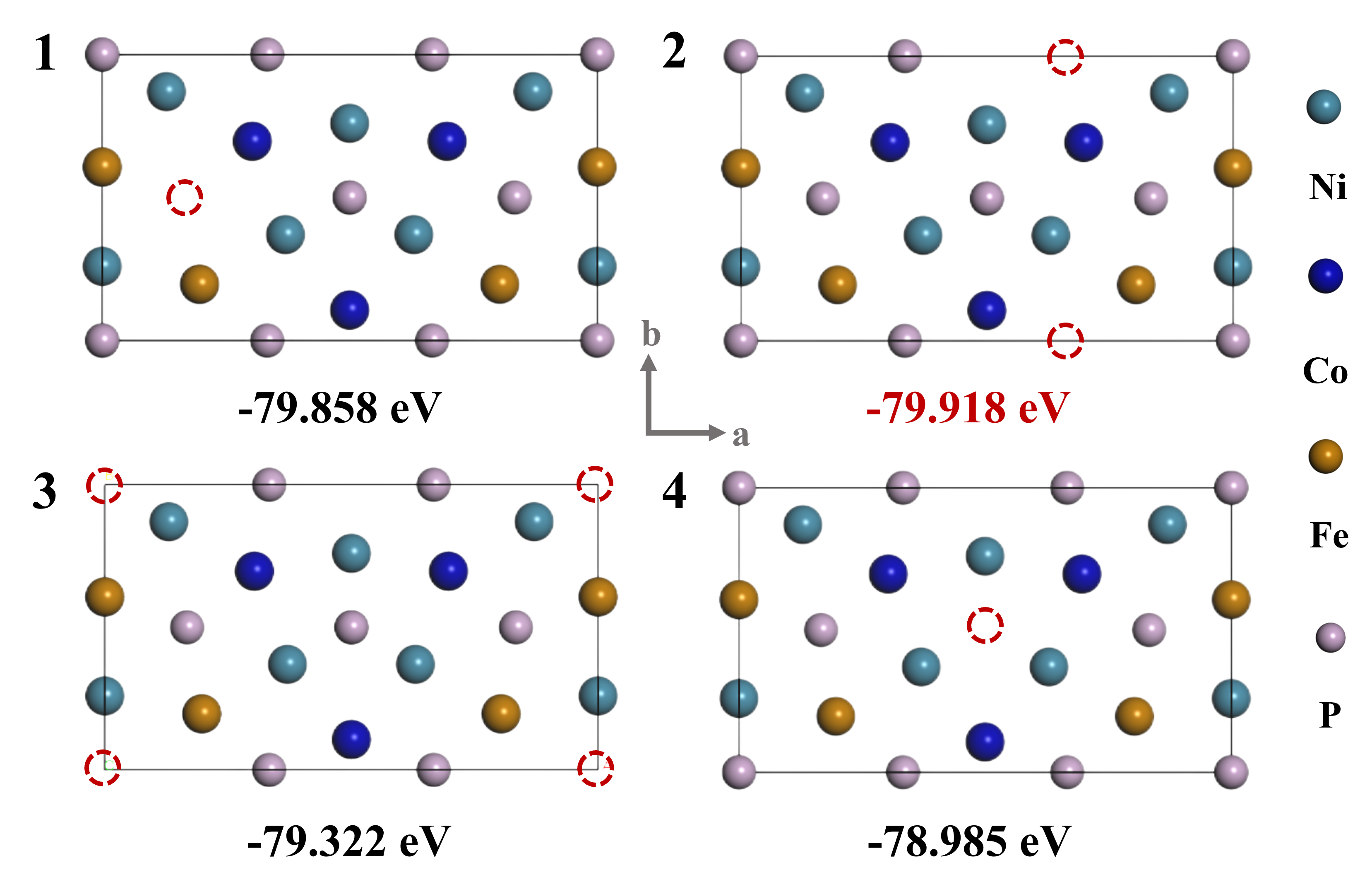


**Figure S7.** Theoretical structure models of NiCo_0.5_Fe_0.5_P with one P vacancy (NiCo_0.5_Fe_0.5_P_1-x_-V_P1_).


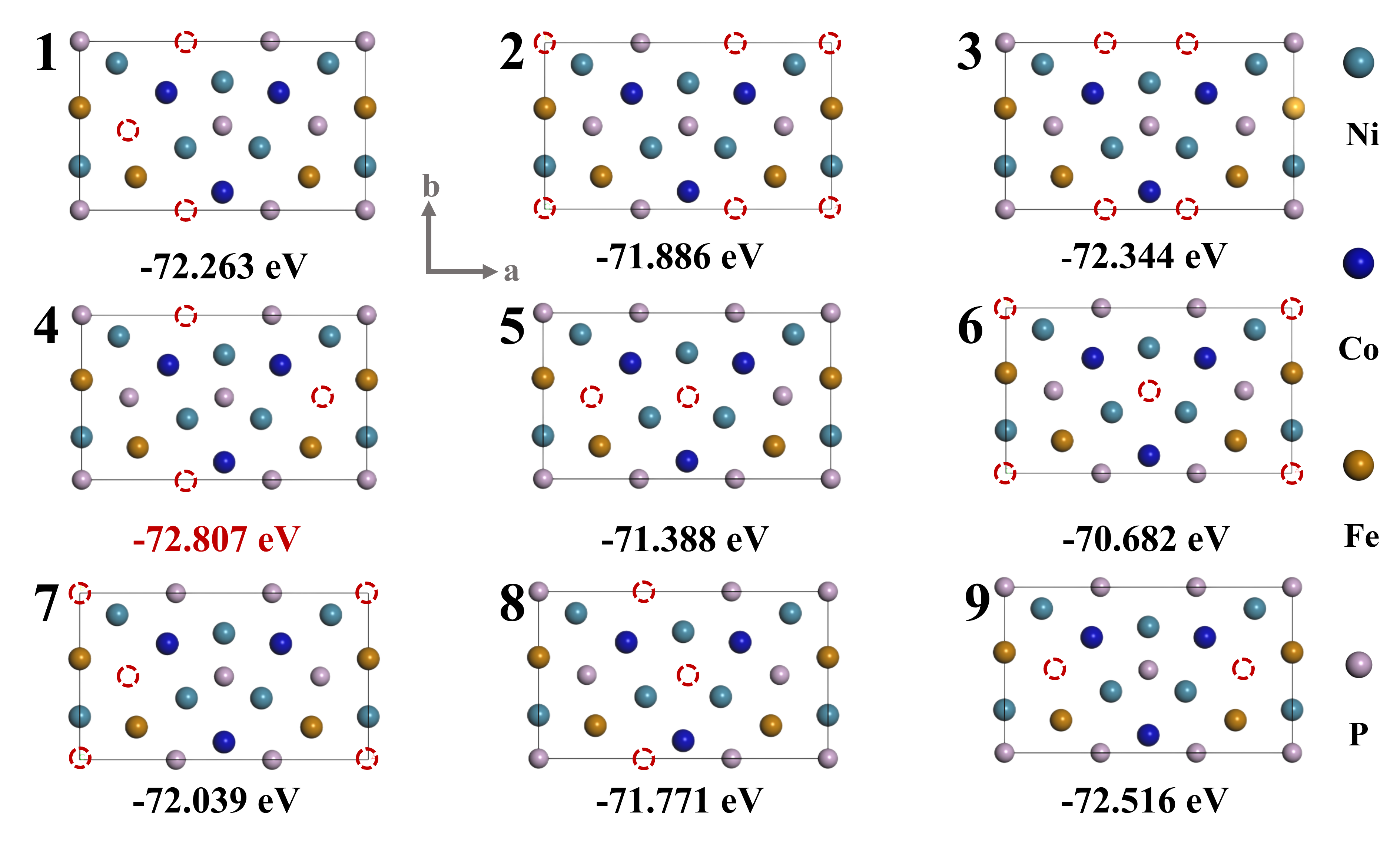


**Figure S8.** Theoretical structure models of NiCo_0.5_Fe_0.5_P with two P vacancies (NiCo_0.5_Fe_0.5_P_1-x_-V_P2_).

**
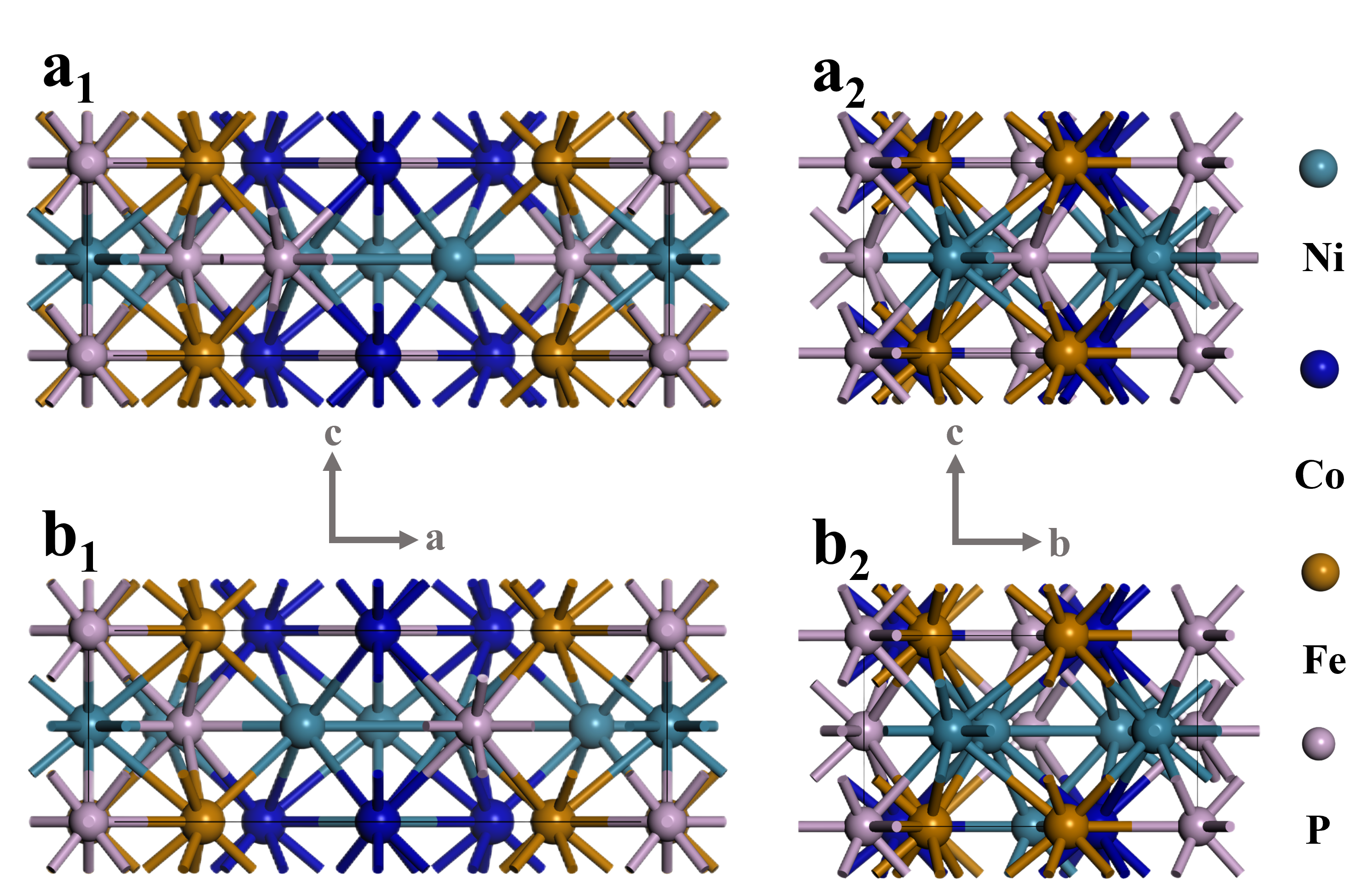
**

**Figure S9.** Theoretical structure models of a) NiCo_0.5_Fe_0.5_P_1-x_-V_P1_ and b) NiCo_0.5_Fe_0.5_P_1-x_-V_P2_ with various orientations.


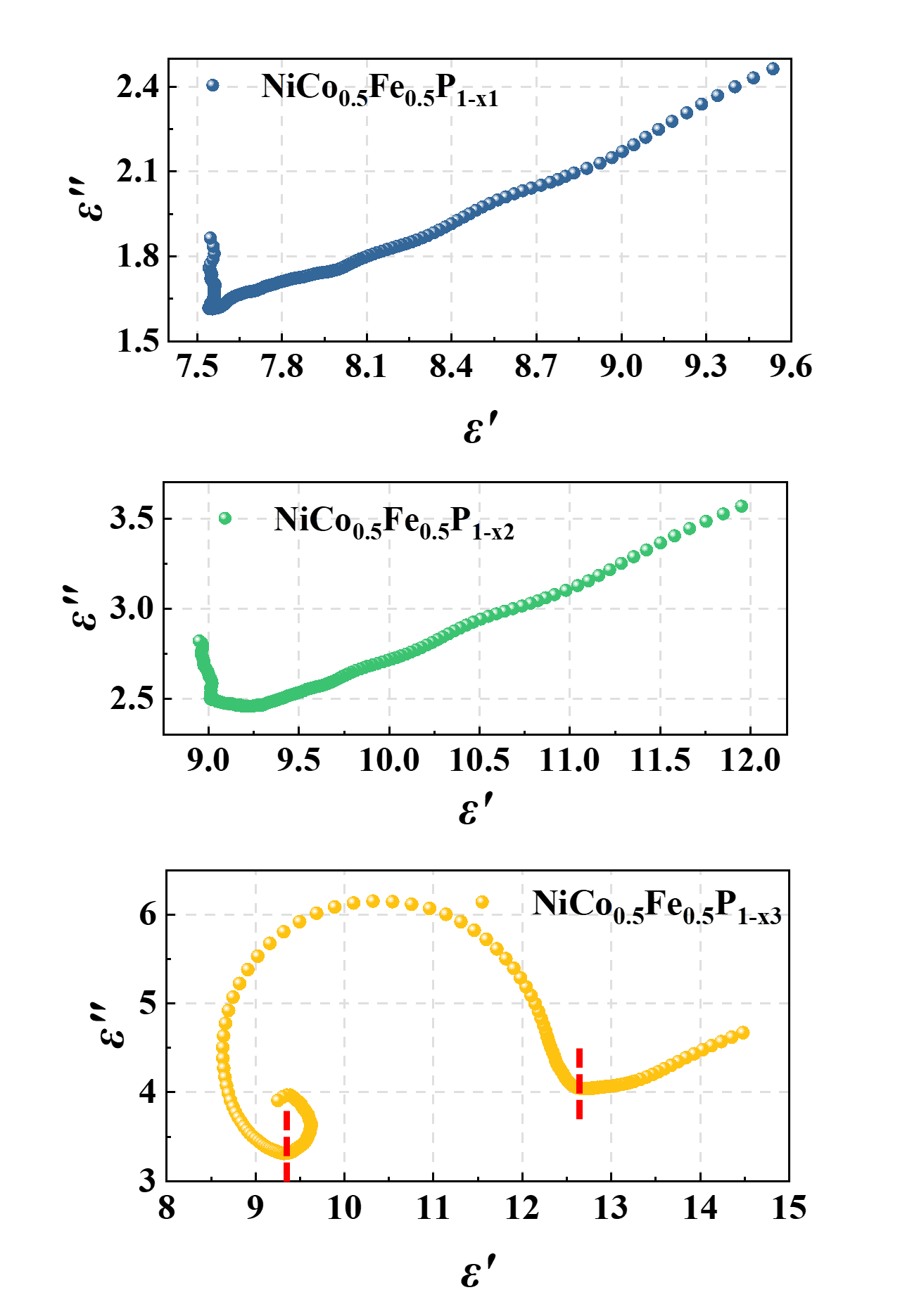


**Figure S10.** Relationship between *ε'* and *ε''* of NiCo_0.5_Fe_0.5_P_1-x_ with various V_P_ contents.


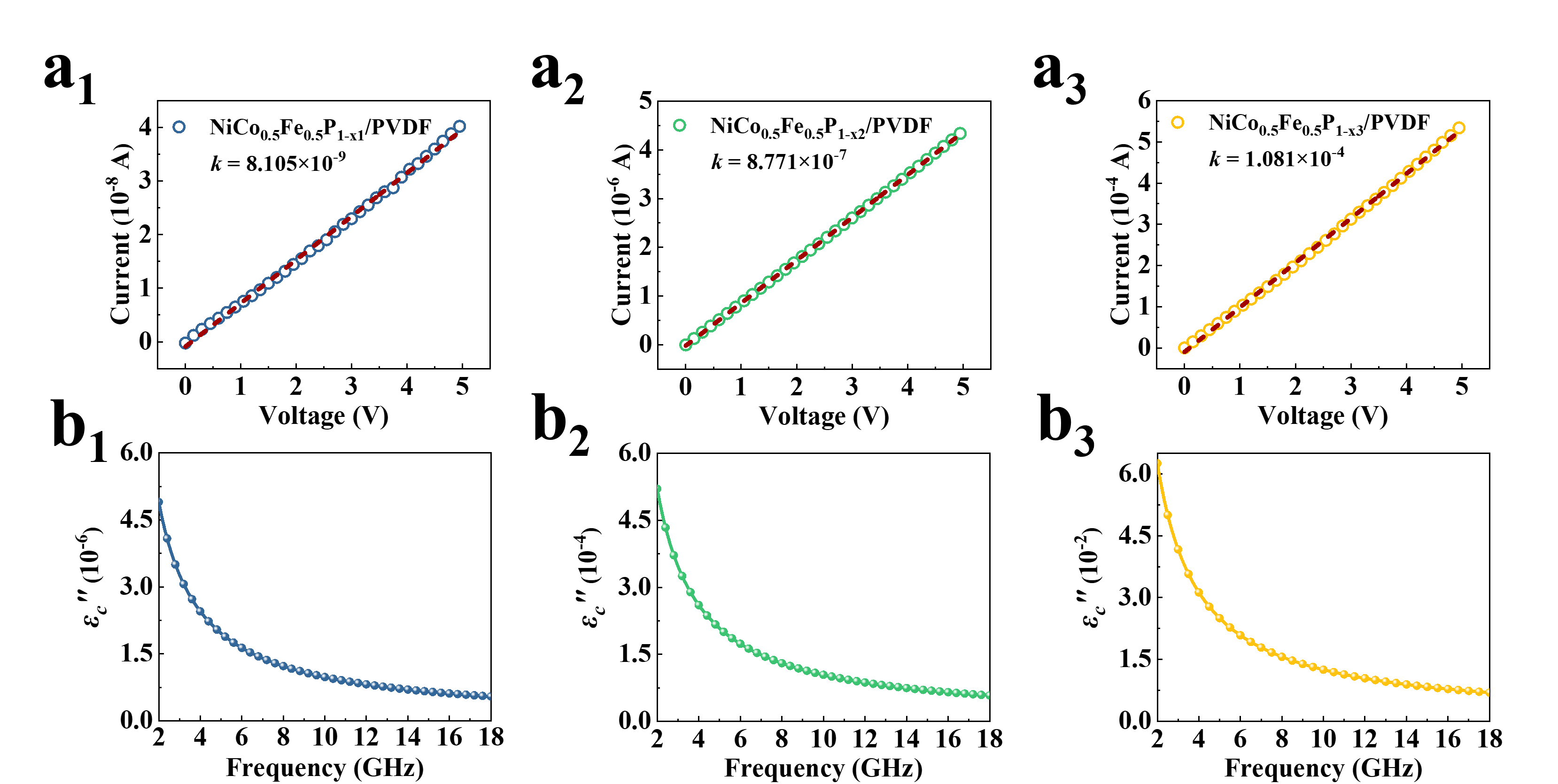


**Figure S11.** U-I curves and conductive loss *ε_c_″*of a_1_/b_1_) NiCo_0.5_Fe_0.5_P_1-x1_/PVDF, a_2_/b_2_) NiCo_0.5_Fe_0.5_P_1-x2_/PVDF, and a_3_/b_3_) NiCo_0.5_Fe_0.5_P_1-x3_/PVDF.


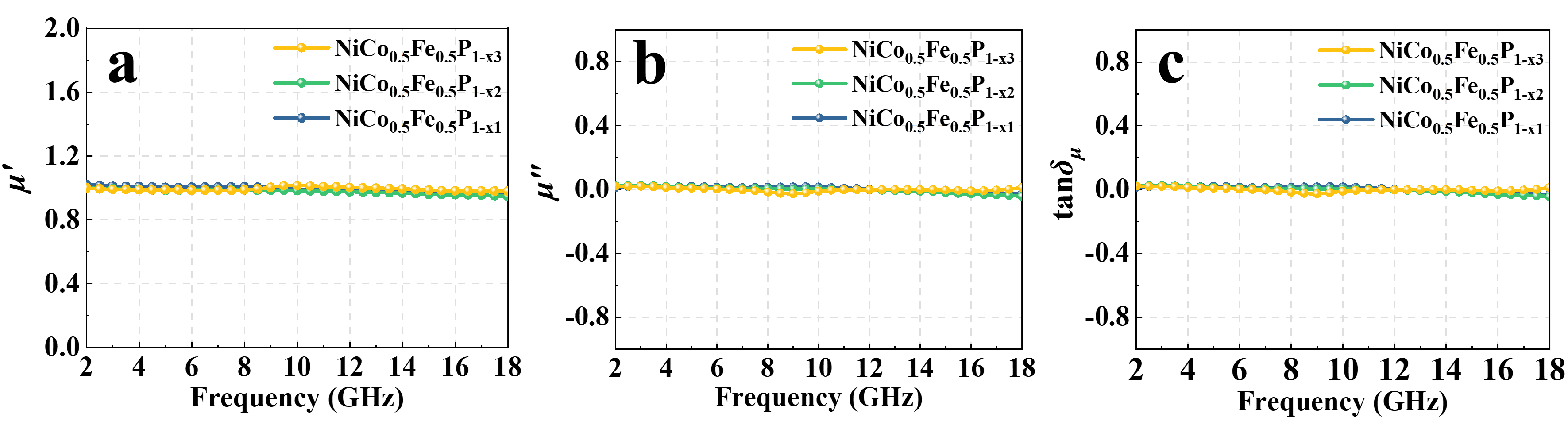


**Figure S12.** Magnetic characterization: a) real part *μ'*, b) imaginary part *μ''*, and c) magnetic loss tangent tan*δ_μ_*.


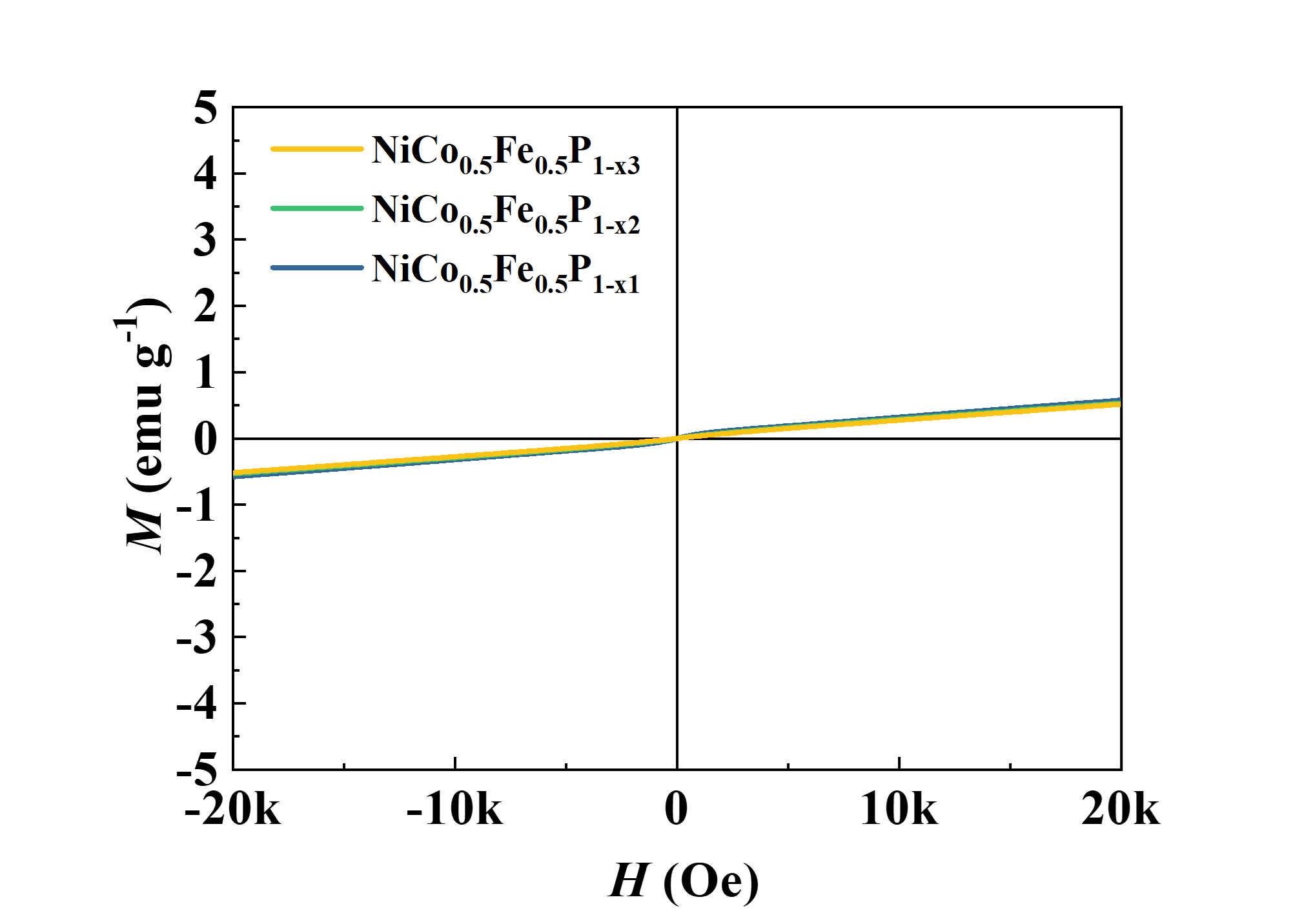


**Figure S13.** Magnetism of NiCo_0.5_Fe_0.5_P_1-x_ with various V_P_ contents.


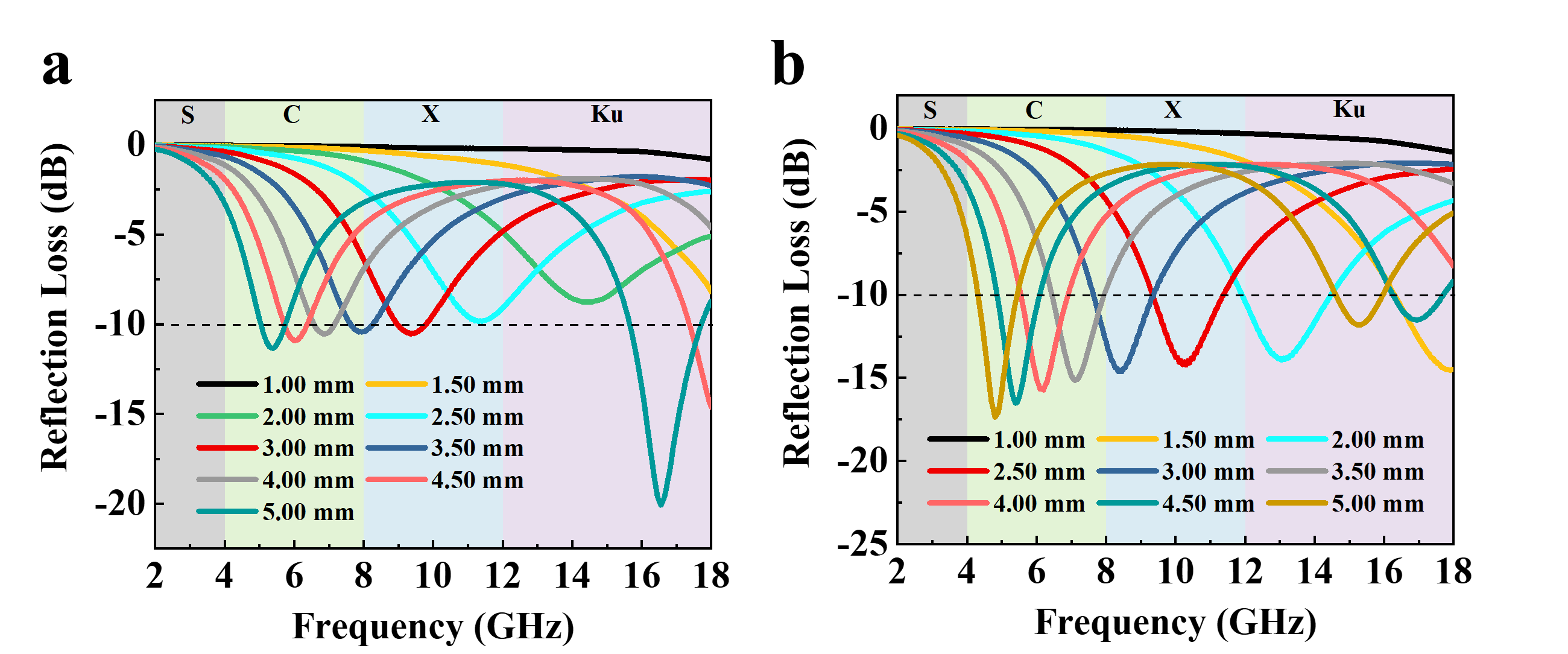


**Figure S14.** 2D RL of a) NiCo_0.5_Fe_0.5_P_1-x1_ and b) NiCo_0.5_Fe_0.5_P_1-x2_.


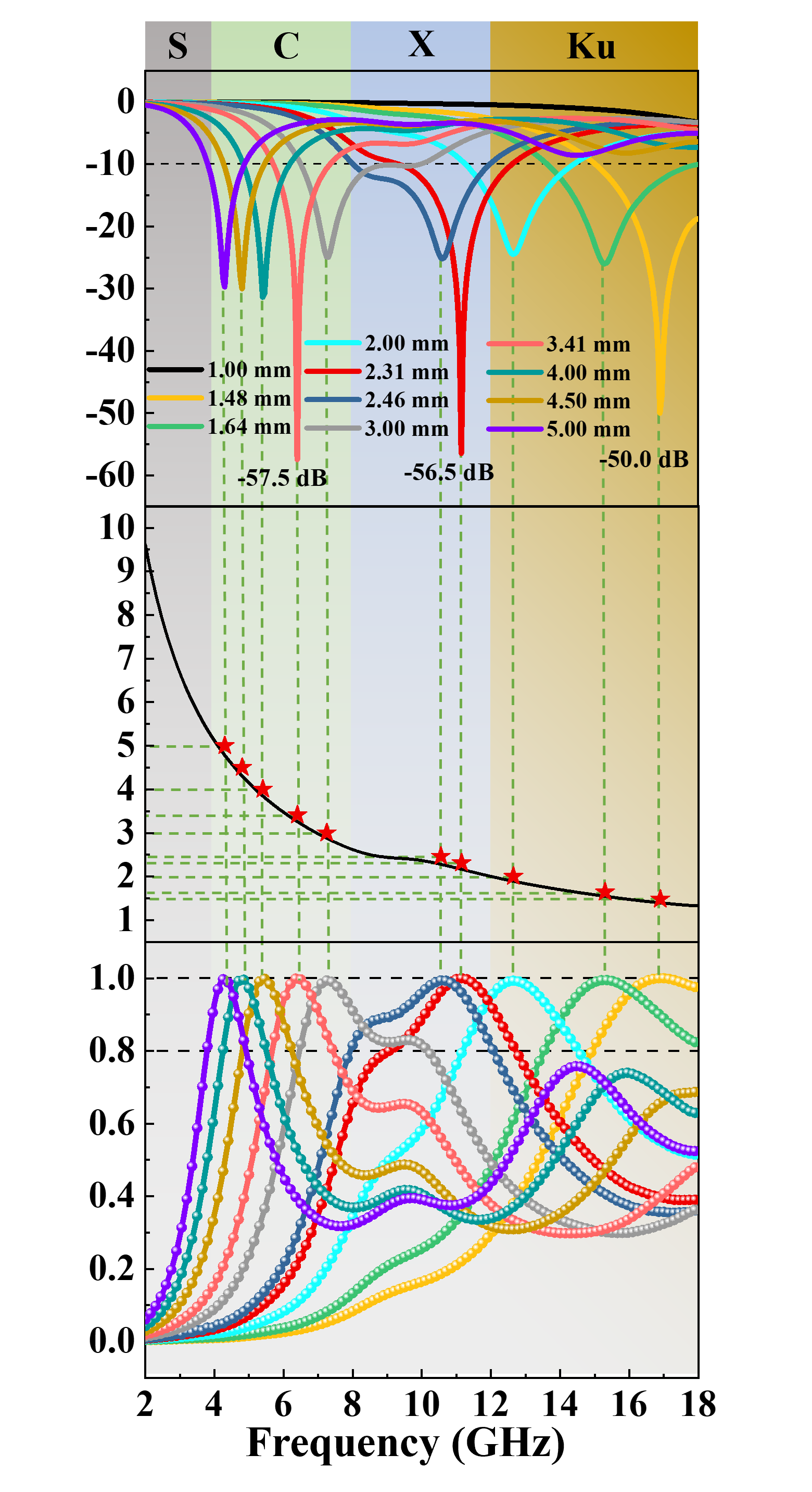


**Figure S15.** 2D RL, dependence of *t_m_* on *f_m_* with the λ/4 model, and *M*_z_ with various thicknesses for NiCo_0.5_Fe_0.5_P_1-x3_.


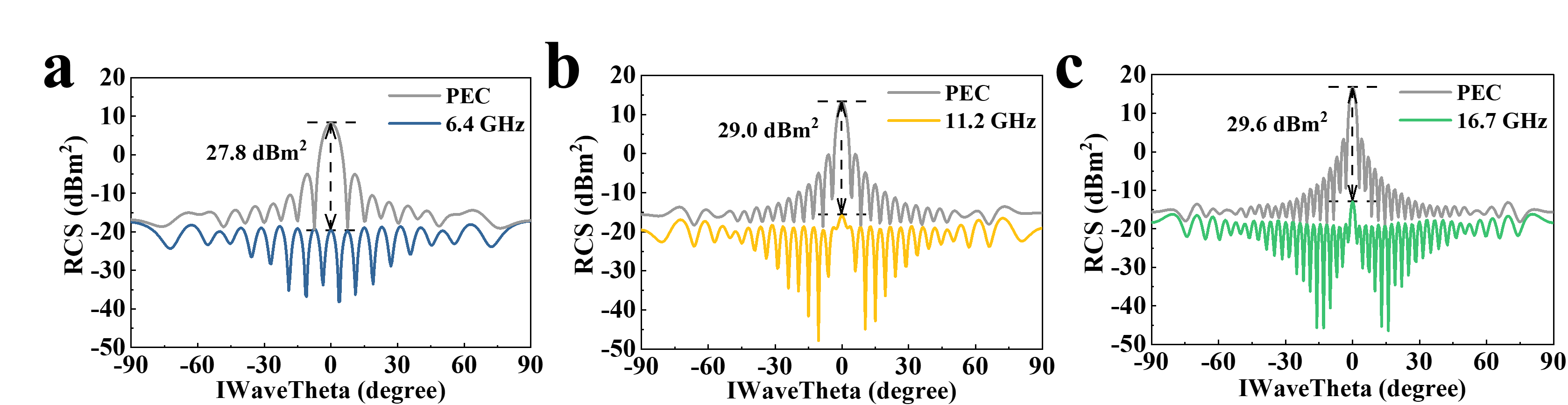


**Figure S16.** RCS simulation curves of NiCo_0.5_Fe_0.5_P_1-x3_ in rectangular coordinates at a) 6.4 GHz/3.41 mm, b) 11.2 GHz /2.31 mm, and c) 16.7 GHz/1.48 mm, respectively.


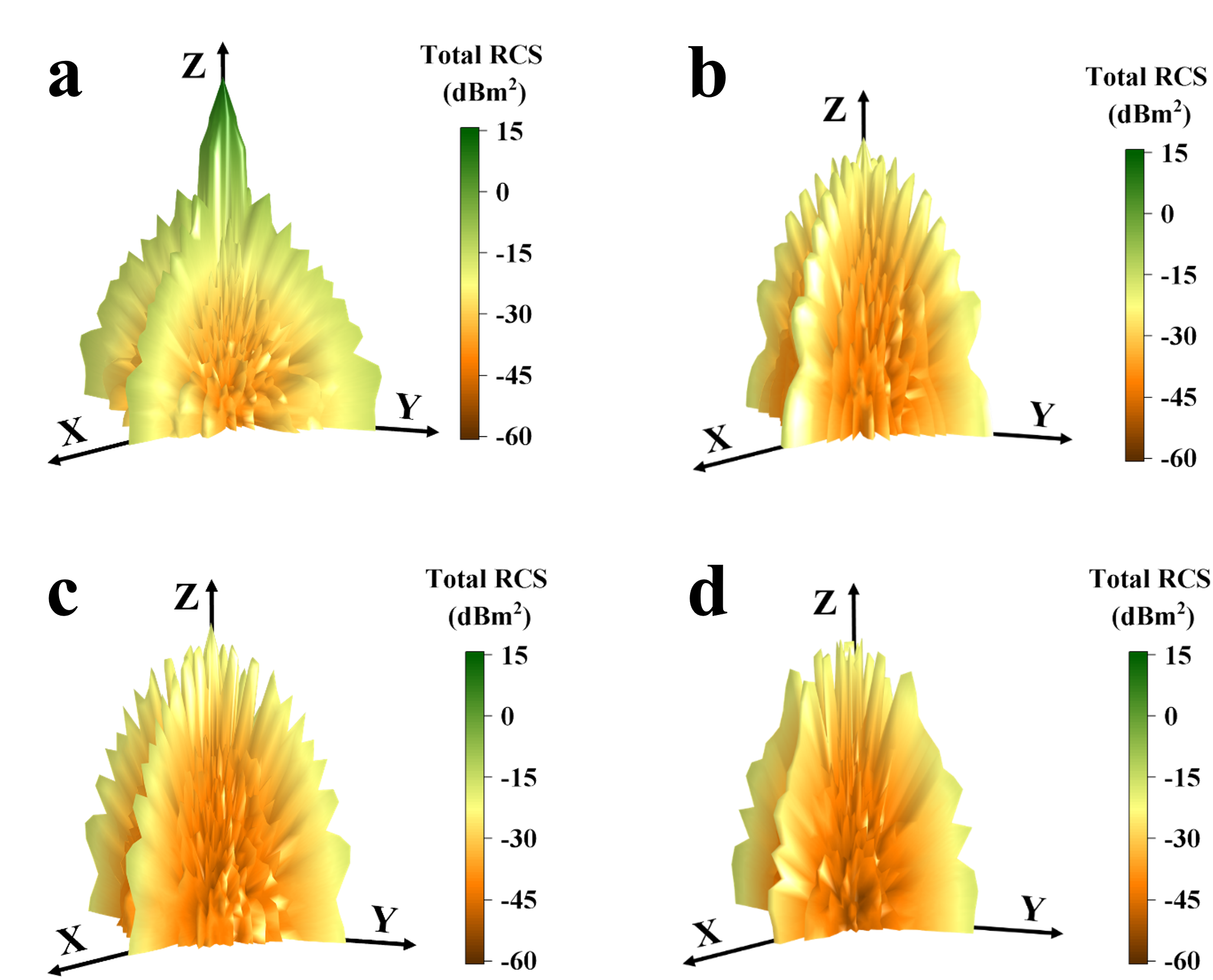


**Figure S17.** RCS 3D radiation patterns of a) PEC and NiCo_0.5_Fe_0.5_P_1-x3_ at b) 6.4 GHz/3.41 mm, c) 11.2 GHz /2.31 mm, and d) 16.7 GHz/1.48 mm.

**Table S1** Valence electron shielding constants for some nonmetallic atoms of groups VA, ⅥA, and ⅦA.

| **ⅤA** | | **ⅥA** | | **ⅦA** | |
| --- | --- | --- | --- | --- | --- |
| N | 3.40 | O | 3.75 | F | 4.10 |
| P | 10.20 | S | 10.55 | Cl | 10.90 |
| As | 26.70 | Se | 27.05 | Br | 27.40 |

**Table S2** Valence electron energy (eV) for some nonmetallic atoms of groups VA, ⅥA, and ⅦA.

| **ⅤA** | | **ⅥA** | | **ⅦA** | |
| --- | --- | --- | --- | --- | --- |
| N | -44.06 | O | -61.41 | F | -81.63 |
| P | -34.82 | S | -44.88 | Cl | -56.23 |
| As | -33.74 | Se | -41.06 | Br | -49.10 |

**Table S3** The metal contents detected by ICP (proportion of single metals to total metals).

| Materials/Ratio | | **Ni** | **Co** | **Fe** |
| --- | --- | --- | --- | --- |
| NiCo_0.5_Fe_0.5_P_1-x1_ | wt.% | 48.62 | 27.95 | 23.43 |
|  | at% | 48.02 | 27.60 | 24.38 |
| NiCo_0.5_Fe_0.5_P_1-x2_ | wt.% | 48.67 | 28.57 | 22.76 |
|  | at% | 48.08 | 28.23 | 23.69 |
| NiCo_0.5_Fe_0.5_P_1-x3_ | wt.% | 49.23 | 28.77 | 22.00 |
|  | at% | 48.66 | 28.42 | 22.92 |

**Table S4** The U-I curve slope, resistance, and conductivity values of coaxial rings.

| **Coaxial rings** | **Thickness (mm)** | ***k***  **(1/Ω)** | ***R* = 1/*k***  **(Ω)** | ***σ***  **(S·m^-1^)** |
| --- | --- | --- | --- | --- |
| NiCo_0.5_Fe_0.5_P_1-x1_/PVDF | 2.10 | 8.105×10^-9^ | 1.234×10^8^ | 5.450×10^-7^ |
| NiCo_0.5_Fe_0.5_P_1-x2_/PVDF | 2.06 | 8.771×10^-7^ | 1.140×10^6^ | 5.787×10^-5^ |
| NiCo_0.5_Fe_0.5_P_1-x3_/PVDF | 2.01 | 1.081×10^-4^ | 9.251×10^3^ | 6.958×10^-3^ |

**Table S5** MA properties of previously reported absorbers and this work.

| Sample | Matrix | Filler content (wt.%) | | RL_min_  /Thickness (dB/mm) | EAB around X  /Thickness  (GHz/mm) | Reference |
| --- | --- | --- | --- | --- | --- | --- |
|  |  |  |  |  |  |  |
| NFO@BFO@PPy | PVDF | 15 | | -65.3 (4.43) C  -34.6 (3.00) X  -16.7(2.00) Ku | 3.7 (3.00) | [6] |
| MoSe_2_/MoC/PNC | wax | 27.5 | | -54.5 (4.10) C  -58.0 (2.40) X  -59.1 (1.90) Ku | 4.4 (2.40) | [45] |
| CoC@FeNiG-F | wax | 10 | -20.0 (3.50) C  -45.0 (2.20) X  -25.0 (1.70) Ku | | 3.9 (2.20) | [46] |
| 1T/2H-MoS_2_ | wax | 55 | -46.0 (4.19) C  -60.0 (2.68) X  -21.0 (2.00) Ku | | 4.0 (3.05) | [47] |
| CoO/CuCo_2_O_4_  /Mxene | PVDF | 10 | -37.0 (3.50) C  -41.0 (3.00) X  -52.7 (1.90) Ku | | 3.3 (2.50) | [48] |
| Fe_3_O_4_-Fe@CNFs  /Al-Fe_3_O_4_-Fe | wax | 30 | -18.0 (2.00) C  -23.0 (3.00) X  -59.3 (4.30) Ku | | 4.0 (3.00) | [49] |
| NiCo_0.5_Fe_0.5_P_1-x3_ | PVDF | 20 | -57.5 (3.41) C  -56.5 (2.31) X  -50.0 (1.48) Ku | | 4.0 (2.46) | This work |

**References**

[S1] X. L, H. Cheng, Y. Li, Q. Chen, C. Liu, C. Shen, X. Liu, *Small* **2024**, *21*, 2407337.
